# Unveiling the future of COVID-19 patient care: groundbreaking prediction models for severe outcomes or mortality in hospitalized cases

**DOI:** 10.3389/fmed.2023.1289968

**Published:** 2024-01-05

**Authors:** Nguyen Thi Kim Hien, Feng-Jen Tsai, Yu-Hui Chang, Whitney Burton, Phan Thanh Phuc, Phung-Anh Nguyen, Dorji Harnod, Carlos Shu-Kei Lam, Tsung-Chien Lu, Chang-I Chen, Min-Huei Hsu, Christine Y. Lu, Chih-Wei Huang, Hsuan-Chia Yang, Jason C. Hsu

**Affiliations:** ^1^Master Program in Global Health and Health Security, College of Public Health, Taipei Medical University, Taipei, Taiwan; ^2^Ph.D. Program in Global Health and Health Security, College of Public Health, Taipei Medical University, Taipei, Taiwan; ^3^PharmD Program, Division of Clinical Pharmacy, College of Pharmacy, Taipei Medical University, Taipei, Taiwan; ^4^International Ph.D. Program in Biotech and Healthcare Management, College of Management, Taipei Medical University, Taipei, Taiwan; ^5^Clinical Data Center, Office of Data Science, Taipei Medical University, Taipei, Taiwan; ^6^Clinical Big Data Research Center, Taipei Medical University Hospital, Taipei Medical University, Taipei, Taiwan; ^7^Research Center of Health Care Industry Data Science, College of Management, Taipei Medical University, Taipei, Taiwan; ^8^Department of Emergency, College of Medicine, Taipei Medical University, Taipei, Taiwan; ^9^Department of Emergency and Critical Care Medicine, Shuang Ho Hospital, Taipei Medical University, New Taipei City, Taiwan; ^10^Division of Emergency, Department of Emergency and Critical Care Medicine, Wan Fang Hospital, Taipei Medical University, Taipei, Taiwan; ^11^Graduate Institute of Injury Prevention and Control, College of Public Health, Taipei Medical University, Taipei, Taiwan; ^12^Department of Emergency Medicine, National Taiwan University Hospital, Taipei, Taiwan; ^13^Department of Healthcare Administration, School of Management, Taipei Medical University, Taipei, Taiwan; ^14^Graduate Institute of Data Science, College of Management, Taipei Medical University, Taipei, Taiwan; ^15^Department of Population Medicine, Harvard Medical School and Harvard Pilgrim Health Care Institute, Boston, MA, United States; ^16^School of Pharmacy, Faculty of Medicine and Health, The University of Sydney, Sydney, NSW, Australia; ^17^Kolling Institute, Faculty of Medicine and Health, The University of Sydney and the Northern Sydney Local Health District, Sydney, NSW, Australia; ^18^International Center for Health Information Technology (ICHIT), Taipei Medical University, Taipei, Taiwan; ^19^Graduate Institute of Biomedical Informatics, College of Medical Science and Technology, Taipei Medical University, Taipei, Taiwan; ^20^Research Center of Big Data and Meta-analysis, Wanfang Hospital, Taipei Medical University, Taipei, Taiwan

**Keywords:** COVID-19, severity, prediction model, Taipei Medical University Clinical Research Database, artificial intelligence, machine learning

## Abstract

**Background:**

Previous studies have identified COVID-19 risk factors, such as age and chronic health conditions, linked to severe outcomes and mortality. However, accurately predicting severe illness in COVID-19 patients remains challenging, lacking precise methods.

**Objective:**

This study aimed to leverage clinical real-world data and multiple machine-learning algorithms to formulate innovative predictive models for assessing the risk of severe outcomes or mortality in hospitalized patients with COVID-19.

**Methods:**

Data were obtained from the Taipei Medical University Clinical Research Database (TMUCRD) including electronic health records from three Taiwanese hospitals in Taiwan. This study included patients admitted to the hospitals who received an initial diagnosis of COVID-19 between January 1, 2021, and May 31, 2022. The primary outcome was defined as the composite of severe infection, including ventilator use, intubation, ICU admission, and mortality. Secondary outcomes consisted of individual indicators. The dataset encompassed demographic data, health status, COVID-19 specifics, comorbidities, medications, and laboratory results. Two modes (full mode and simplified mode) are used; the former includes all features, and the latter only includes the 30 most important features selected based on the algorithm used by the best model in full mode. Seven machine learning was employed algorithms the performance of the models was evaluated using metrics such as the area under the receiver operating characteristic curve (AUROC), accuracy, sensitivity, and specificity.

**Results:**

The study encompassed 22,192 eligible in-patients diagnosed with COVID-19. In the full mode, the model using the light gradient boosting machine algorithm achieved the highest AUROC value (0.939), with an accuracy of 85.5%, a sensitivity of 0.897, and a specificity of 0.853. Age, vaccination status, neutrophil count, sodium levels, and platelet count were significant features. In the simplified mode, the extreme gradient boosting algorithm yielded an AUROC of 0.935, an accuracy of 89.9%, a sensitivity of 0.843, and a specificity of 0.902.

**Conclusion:**

This study illustrates the feasibility of constructing precise predictive models for severe outcomes or mortality in COVID-19 patients by leveraging significant predictors and advanced machine learning. These findings can aid healthcare practitioners in proactively predicting and monitoring severe outcomes or mortality among hospitalized COVID-19 patients, improving treatment and resource allocation.

## Introduction

The emergence of the coronavirus disease 2019 (COVID-19) outbreak in China during late 2019 has escalated into a worldwide health apprehension, primarily due to its rapid transmission and deleterious health implications (1). Its prevalent symptoms encompass fever, dry cough, and dyspnea (2). According to prior investigations, a distinct subset of afflicted individuals faces a heightened susceptibility to severe infection, with respiratory impairments such as dyspnea, elevated respiratory rate, and diminished oxygen saturation dominating the symptomatology. Individuals with advanced disease may also manifest respiratory failure, septic shock, or multi-organ dysfunction (3).

The swift propagation and extensive ramifications of this worldwide pandemic have imposed a significant strain on healthcare systems across diverse nations. This strain is particularly evident in the realms of clinical resource allocation and decision-making protocols. Numerous medical institutions have encountered unparalleled scarcities of essential supplies, among them mechanical ventilators, primarily stemming from the rapid surge in critically ill COVID-19 patients necessitating both airway assistance and mechanical ventilatory support. This predicament, confronting healthcare delivery systems, underscores the urgency of employing innovative and pioneering technologies to navigate acute and systemic challenges in healthcare provisioning. With the overarching aims of mitigating mortality and sustaining healthcare infrastructure, the primary objective entails averting severe outcomes and fatalities among patients.

The incorporation of artificial intelligence (AI) and machine learning (ML) within the healthcare domain, spanning tasks such as image analysis, clinical decision-making, and prognosis prediction, constitutes a burgeoning discipline with broad applications across diverse maladies (4). Within the context of COVID-19, artificial intelligence has demonstrated its pivotal role in both diagnostic and prognostic domains, encompassing prediction, detection, classification, screening, and diagnosis of COVID-19 infections (5, 6). Scoping reviews have underscored the potential of artificial intelligence as a weapon in the fight against COVID-19; nonetheless, many proposed methodologies are yet to secure clinical acceptance (7). Predictive models stand as extensively investigated tools within biotechnology, enriching clinical comprehension of the diagnostic and prognostic dimensions of various illnesses.

According to the Taiwan Centers for Disease Control, during the initial phase of the COVID-19 outbreak, a substantial proportion (42%) of the cases were primarily located in the northern region of Taiwan, probably due to the presence of the International airports in that area and May 2022 marked the onset of the first wave of the pandemic (8). The Taipei Medical University Clinical Research Database (TMUCRD) gathers data from multiple centers and sources of various data types. It systematically collects both structured and unstructured data from three affiliated hospitals: Taipei Medical University Hospital, Wanfang Hospital, and Shuangho Hospital (9–11). The National Health Insurance database in Taiwan has a gap of 2 years in the dissemination of data for research purposes. Therefore, in terms of finding recent breakthroughs in the field of COVID-19, TMUCRD could help enhance the understanding of factors influencing COVID-19 outcomes.

Based on the most accurate information available, no prediction model study of COVID-19 severe symptoms in Taiwan. This study aimed to predict severe outcomes, including the use of ventilators, intubation, admission to the intensive care unit (ICU), and mortality, among COVID-19 patients hospitalized in Taiwan. The primary objective of this study is to develop predictive models that can assist clinicians in identifying individuals who are most vulnerable to severe outcomes, including mortality. This focused identification provides healthcare practitioners with the tools to carry out prompt interventions.

## Methods

### Study design and data source

To create the dataset, this study utilized clinical data obtained from the Taipei Medical University Clinical Research Database (TMUCRD). TMUCRD consolidates extensive clinical data derived from three associated hospitals: Taipei Medical University Hospital, Wanfang Hospital, and Shuang-Ho Hospital. The database comprises structured and unstructured information. This study obtained approval from the Taipei Medical University Joint Institutional Review Board (TMU-JIRB) with grant number N202302020.

### Population selection

This study included patients who were hospitalized and confirmed to have contracted COVID-19 within the period spanning from January 1, 2021, to May 31, 2022. The diagnosis of COVID-19 was established either through a positive outcome from a real-time reverse transcription polymerase chain reaction (RT-PCR) test or a positive outcome from a rapid antigen test.

**Figure 1:**
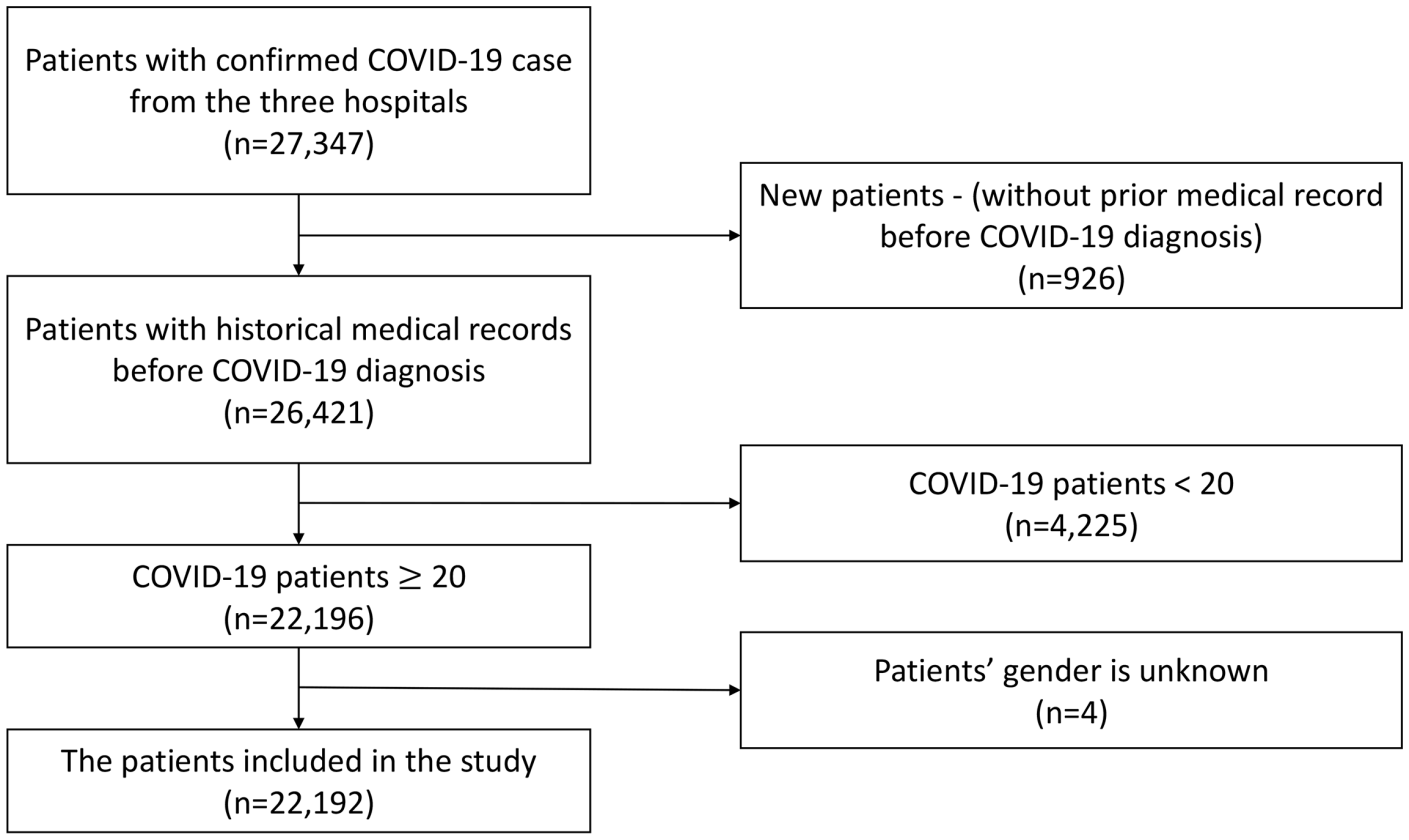
Flowchart of cohort selection.

The exclusion criteria encompassed newly registered patients who had not previously sought medical care at the three hospitals due to the lack of complete medical background information records, individuals under the age of 20, and patients with undisclosed gender information. As a result, a total of 22,192 patients were retained for inclusion in this study. The selection process for the study population is visually depicted in Figure 1.

### Outcome measurement

The index date is defined as the date of the first COVID diagnosis. The primary outcome was defined as a serious event, encompassing occurrences such as ventilator use, intubation, intensive care unit (ICU) admission, and mortality within 3 months of confirmed COVID-19 infection. Additionally, each of the aforementioned specific indicators was considered as a secondary outcome in this study. Data censoring occurred either at the date of death, loss to follow-up, or at the end of the study (May 31, 2022).

### Features

Based on a literature review and consultation with clinicians, this study identified features associated with the above outcomes based on demographic information, health status, COVID-19-related details, comorbidities, long-term medication records, and laboratory test results. The selected features include: (1) demographic information: gender and age; (2) health status: body mass index (BMI) and Charlson Comorbidity Index (CCI) score; (3) COVID-19-related details: COVID-19 vaccine and Covid-19 medications; (4) comorbidities: myocardial infarction (MI), chronic kidney disease (CKD), congestive heart failure (CHF), peripheral vascular disease, cerebrovascular disease (CVA), cardiovascular disease (CVD), dementia, chronic obstructive pulmonary disease (COPD), rheumatic disease, peptic ulcer disease, liver disease, diabetes mellitus (DM), hemiplegia, renal disease, cancer, human immunodeficiency virus/ acquired immune deficiency syndrome (HIV/AIDS), hypertension, hyperlipidemia, hyperuricemia, depression or anxiety, anemia, Parkinson’s disease (PD), osteoporosis; (5) long-term medication records: benzodiazepine (BZD), non-steroidal anti-inflammatory drug (NSAID), aspirin, hypertension (HTN) drugs, DM drugs, statins, antihyperuricemic drugs, antihistamin, gastro-oesophageal reflux disease (GORD) drugs, steroids; and (6) laboratory test results: HbA1C, total cholesterol (TC), high-density lipoprotein (HDL), low-density lipoprotein (LDL), triglycerides (TG), Uric acid (UA), aspartate aminotransferase/AST (GOT), alanine transaminase/ALT (GPT), total protein, albumin, globubin, blood urea nitrogen (BUN), creatinine, red blood cells (RBC), hemoglobin (HGB), mean corpuscular hemoglobin (MCH), mean corpuscular hemoglobin concentration (MCHC), white blood cell (WBC), neutrophil, lymphocyte, platelet count (PLT), hematocrit (HCT), sodium (NA), potassium (K), troponin I, and troponin T.

The Charlson Comorbidity Index (CCI) score was computed, and comorbidity was determined using disease codes sourced from the ICD-9 or ICD-10 classification systems found in the medical records. Among the cohort members, individuals were categorized as having comorbidities if they had undergone a minimum of two outpatient visits or one hospitalization related to the specific disease before the index date. Evaluation of the COVID-19 vaccine status is based on the vaccination records within the year preceding the index date. Assessment of COVID-19 medications is grounded in the medication status during the 3 months following the index date. Long-term medication users in the cohort were characterized as patients who had received a prescription for one or more of the aforementioned drugs for a period of 28 days or longer in the year (365 days) prior to the index date. In cases where multiple test results were obtainable, priority was given to the latest laboratory test value within a one-year period before the index date. The technique of Multiple Imputation by Chained Equations (MICE) was employed to address the presence of missing continuous features (12).

### Statistical analysis

In the realm of descriptive statistics, continuous data are elucidated through the utilization of the mean (standard deviation, S.D.) and median (minimum and maximum values). Conversely, categorical data are expounded upon by presenting the count of cases along with their corresponding percentages. Additionally, the count and proportion of missing values were computed. Statistical analyses were conducted employing R version 4.1.3 (R Project for Statistical Computing).

### Algorithms used in this study

Seven machine learning algorithms were utilized to formulate personalized prediction models. The machine learning algorithms encompass Linear Discriminant Analysis (LDA), Logistic Regression (LR), Support Vector Machine (SVM), Random Forest (RF), Gradient Boosting Machine (GBM), Light GBM, and Extreme Gradient Boosting (XGBoost) (refer to Supplementary Appendix 1). Prediction models were developed in this study based on two modes and employing diverse algorithms: (1) Full mode: encompassing all selected features’ data; (2) Simplified mode: incorporating 30 crucial features chosen based on the algorithm used by the best model in full mode.

### Model training and testing

The participant cohort was divided into training and testing datasets, with 80% of participants assigned to the training subset, and the remaining portion constituting the testing dataset. The cross-validation technique was also performed to access the over-fitting (13, 14).

### Evaluation of model performance and interpretation

Performance assessment and comparison of all prediction models involved the calculation of metrics including the area under the receiver operating characteristic curve (AUROC), accuracy, sensitivity (recall), specificity, positive predictive value (PPV or precision), negative predictive value (NPV), and F1-score. The optimal model was determined by identifying the one with the highest AUROC through a comparative analysis of various models using testing results. Data processing was executed using MSSQL Server 2017, while model training and testing were carried out utilizing the Python programming language version 3.9 (15). The SHapley Additive exPlanations (SHAP) values were used to assess feature’s contribution (also known as its importance) to the most optimal model when interpreting the models (16).

## Results

### Baseline of patient characteristics

Table 1 shows basic characteristics of the study cohort, including patients’ demographic information, health status, COVID-19-related details, comorbidities, long-term medication records, and laboratory test results. In this study, 22,192 inhospitalized patients were included. Among the entire patient cohort, there were 12,452 female patients (56.1%), slightly outnumbering the 9,740 male patients (43.9%). The patients had a mean age of 49.3 (S.D. 17.4), with the majority falling below 65 years old (17,625, 79.4%), followed by those aged 65–85 (3,960, 17.8%), and those above 85 (607, 2.7%). Among the subset of patients with available BMI records (11,695), the patients’ average BMI was 24.4 (S.D. 4.51). The majority had a BMI greater than or equal to 24 (48.32%), while 45.44% had BMIs between 18.5 and 24, and 6.24% had BMIs below 18.5. The patients had an average CCI score of 0.53 (S.D. 1.52), with the majority achieving a CCI score of 0 (18,298, 82.5%). Following were patients with CCI scores ranging from 0 to 3 (2,115, 9.5%), while a smaller portion exhibited scores greater than 3 (1,779, 8.0%). A total of 5,820 individuals (26.2% of all patients) had a history of vaccine succession, while 558 individuals (2.5% of all patients) had received anti-COVID-19 virus drugs (Paxlovid or Molnupiravir). Complete basic patient characteristics are provided in Appendix 2.

**Table 1:** Baseline of patient characteristics.

Variables	Total (*N* = 22,192)	Ventilator (*N* = 1,010)	Intubation (*N* = 196)	ICU (*N* = 85)	Mortality (*N* = 205)
Demographic information					
Sex, *N* (%)					
Female	12,452 (56.1%)	459 (45.4%)	84 (42.9%)	28 (32.9%)	72 (35.1%)
Male	9,740 (43.9%)	551 (54.6%)	112 (57.1%)	57 (67.1%)	133 (64.9%)
Age, *N* (%)					
Mean (SD)	49.3 (17.4)	71.4 (17.2)	66.5 (15.0)	72.9 (13.6)	78.2 (12.4)
Median [Min, Max]	47.4 [20.0, 110]	73.5 [20.0, 108]	69.4 [20.0, 97.7]	72.8 [32.2, 97.7]	79.5 [43.1, 102]
Age < 65 yrs.	17,625 (79.4%)	303 (30.0%)	77 (39.3%)	17 (20.0%)	28 (13.7%)
65 ≤ Age < 85 yrs.	3,960 (17.8%)	459 (45.4%)	100 (51.0%)	52 (61.2%)	110 (53.7%)
Age ≥ 85 yrs.	607 (2.7%)	248 (24.6%)	19 (9.7%)	16 (18.8%)	67 (32.7%)
Health status					
BMI, *N* (%)					
Mean (SD)	24.4 (4.51)	23.8 (4.71)	25.1 (4.83)	24.8 (4.49)	23.1 (4.39)
Median [Min, Max]	23.8 [9.21, 51.9]	23.4 [12.5, 48.5]	24.4 [15.7, 43.8]	24.0 [16.9, 37.8]	22.4 [13.5, 41.6]
BMI < 18.5	730 (3.3%)	95 (9.4%)	14 (7.1%)	4 (4.7%)	21 (10.2%)
18.5 < = BMI < 24	5,314 (23.9%)	397 (39.3%)	63 (32.1%)	36 (42.4%)	96 (46.8%)
BMI > = 24	5,651 (25.5%)	401 (39.7%)	96 (49.0%)	40 (47.1%)	68 (33.2%)
CCI score, *N* (%)					
Mean (SD)	0.530 (1.52)	1.88 (2.68)	1.76 (2.41)	1.89 (2.84)	1.80 (3.08)
Median [Min, Max]	0 [0, 18.0]	0 [0, 16.0]	1.00 [0, 11.0]	0 [0, 16.0]	0 [0, 16.0]
CCI score = 0	18,298 (82.5%)	517 (51.2%)	95 (48.5%)	44 (51.8%)	131 (63.9%)
0 < = CCI score < 3	2,115 (9.5%)	187 (18.5%)	44 (22.4%)	16 (18.8%)	17 (8.3%)
CCI score > = 3	1779 (8.0%)	306 (30.3%)	57 (29.1%)	25 (29.4%)	57 (27.8%)
COVID-19-related details					
COVID-19 vaccine	5,820 (26.2%)	151 (15.0%)	23 (11.7%)	9 (10.6%)	24 (11.7%)
COVID-19 medications (Paxlovid or Molnupiravir)	558 (2.5%)	49 (4.9%)	4 (2.0%)	2 (2.4%)	4 (2.0%)
Comorbidities, *N* (%)					
Congestive heart failure (CHF)	534 (2.4%)	111 (11.0%)	17 (8.7%)	5 (5.9%)	17 (8.3%)
Cardiovascular disease	997 (4.5%)	182 (18.0%)	34 (17.3%)	17 (20.0%)	29 (14.1%)
COPD	1,106 (5.0%)	155 (15.3%)	30 (15.3%)	13 (15.3%)	23 (11.2%)
Peptic ulcer disease	1,367 (6.2%)	153 (15.1%)	30 (15.3%)	14 (16.5%)	26 (12.7%)
Liver disease	860 (3.9%)	85 (8.4%)	22 (11.2%)	9 (10.6%)	22 (10.7%)
Diabetes mellitus	1,347 (6.1%)	210 (20.8%)	31 (15.8%)	17 (20.0%)	41 (20.0%)
Renal disease	673 (3.0%)	131 (13.0%)	26 (13.3%)	13 (15.3%)	29 (14.1%)
Cancer	535 (2.4%)	90 (8.9%)	20 (10.2%)	9 (10.6%)	18 (8.8%)
Hypertension	1,490 (6.7%)	240 (23.8%)	50 (25.5%)	25 (29.4%)	45 (22.0%)
Hyperlipidemia	2055 (9.3%)	213 (21.1%)	52 (26.5%)	23 (27.1%)	31 (15.1%)
Depression or anxiety	884 (4.0%)	87 (8.6%)	18 (9.2%)	6 (7.1%)	9 (4.4%)
Anemia	621 (2.8%)	92 (9.1%)	21 (10.7%)	7 (8.2%)	17 (8.3%)
Long-term medication, *N* (%)					
BZD	1,695 (7.6%)	216 (21.4%)	42 (21.4%)	22 (25.9%)	71 (34.6%)
NSAID	1,016 (4.6%)	65 (6.4%)	20 (10.2%)	10 (11.8%)	16 (7.8%)
Aspirin	1,396 (6.3%)	178 (17.6%)	40 (20.4%)	22 (25.9%)	54 (26.3%)
HTN	2,846 (12.8%)	323 (32.0%)	62 (31.6%)	32 (37.6%)	90 (43.9%)
DM	1,250 (5.6%)	154 (15.2%)	25 (12.8%)	14 (16.5%)	49 (23.9%)
Statin	2,141 (9.6%)	181 (17.9%)	43 (21.9%)	21 (24.7%)	46 (22.4%)
Antihyperuricemic	418 (1.9%)	56 (5.5%)	11 (5.6%)	5 (5.9%)	27 (13.2%)
Antihistamin	528 (2.4%)	49 (4.9%)	7 (3.6%)	4 (4.7%)	14 (6.8%)
GORD	1,317 (5.9%)	182 (18.0%)	35 (17.9%)	21 (24.7%)	54 (26.3%)
Steroids	2,420 (10.9%)	228 (22.6%)	56 (28.6%)	28 (32.9%)	69 (33.7%)
Laboratory test results, *N* (%)					
AST (GOT)					
Mean (SD)	30.2 (123)	53.4 (288)	115 (663)	53.6 (84.3)	113 (597)
Median [Min, Max]	21.0 [0, 7,930]	26.5 [8.00, 7,930]	31.0 [11.0, 7,930]	32.0 [14.0, 553]	35.0 [8.00, 7,930]
ALT (GPT)					
Mean (SD)	26.1 (57.1)	37.2 (150)	57.2 (243)	37.2 (83.0)	63.1 (233)
Median [Min, Max]	19.0 [0, 2,690]	18.0 [0, 2,690]	20.0 [0, 2,580]	20.0 [0, 654]	20.0 [0, 2,580]
Creatinine					
Mean (SD)	1.28 (1.88)	1.87 (2.51)	2.00 (2.45)	2.28 (2.62)	2.16 (2.37)
Median [Min, Max]	0.820 [0, 23.3]	1.00 [0, 19.3]	1.08 [0.340, 17.8]	1.29 [0.340, 17.8]	1.27 [0, 17.8]
RBC					
Mean (SD)	4.39 (0.741)	4.08 (0.860)	4.21 (0.968)	3.95 (0.812)	3.74 (0.912)
Median [Min, Max]	4.44 [1.03, 7.67]	4.11 [1.03, 6.87]	4.34 [1.67, 6.85]	4.02 [2.20, 6.24]	3.75 [1.77, 7.19]
Hemoglobin (HGB)					
Mean (SD)	13.0 (2.04)	12.1 (2.39)	12.4 (2.58)	12.0 (2.39)	11.3 (2.61)
Median [Min, Max]	13.3 [3.40, 25.2]	12.4 [4.50, 18.3]	12.8 [4.70, 17.6]	12.0 [7.00, 16.8]	11.4 [5.80, 16.8]
MCH					
Mean (SD)	29.7 (3.11)	29.9 (3.24)	29.8 (3.59)	30.6 (3.06)	30.3 (3.04)
Median [Min, Max]	30.3 [12.9, 43.4]	30.5 [13.2, 43.4]	30.5 [17.2, 36.7]	30.8 [20.9, 35.3]	30.5 [18.8, 38.4]
MCHC					
Mean (SD)	33.8 (1.20)	33.8 (1.44)	33.6 (1.62)	33.8 (1.23)	33.6 (1.33)
Median [Min, Max]	33.9 [16.6, 40.1]	33.9 [16.6, 40.1]	33.8 [27.2, 40.1]	33.9 [29.7, 36.5]	33.8 [29.6, 37.1]
WBC					
Mean (SD)	7.39 (3.31)	8.14 (4.78)	9.13 (7.30)	9.59 (6.30)	10.3 (8.32)
Median [Min, Max]	6.78 [0.200, 78.7]	7.19 [0.570, 78.7]	7.72 [0.570, 78.7]	8.40 [0.570, 37.8]	8.58 [0.570, 78.7]
Neutrophil					
Mean (SD)	67.4 (13.6)	74.3 (13.4)	74.5 (14.7)	77.2 (12.5)	78.8 (13.9)
Median [Min, Max]	67.4 [0, 99.0]	75.7 [0, 99.0]	76.5 [0, 96.5]	78.2 [34.3, 96.5]	81.5 [0, 98.5]
PLT					
Mean (SD)	230 (80.9)	197 (87.1)	190 (88.1)	175 (97.6)	178 (88.5)
Median [Min, Max]	226 [0, 1,010]	182 [12.0, 652]	172 [14.0, 569]	151 [14.0, 569]	155 [14.0, 478]
HCT					
Mean (SD)	38.3 (5.85)	35.8 (6.91)	36.8 (7.44)	35.5 (6.85)	33.4 (7.68)
Median [Min, Max]	39.1 [10.4, 55.5]	36.8 [11.7, 52.0]	37.9 [11.7, 51.3]	35.8 [22.0, 48.4]	33.4 [16.4, 50.6]
NA					
Mean (SD)	138 (4.52)	135 (6.01)	136 (6.68)	135 (5.48)	137 (7.28)
Median [Min, Max]	138 [68.5, 167]	136 [103, 167]	136 [103, 163]	135 [111, 146]	137 [111, 162]
*K*					
Mean (SD)	4.04 (0.538)	3.98 (0.661)	4.05 (0.762)	4.13 (0.866)	4.11 (0.781)
Median [Min, Max]	4.00 [2.01, 7.50]	3.90 [2.20, 7.50]	3.90 [2.60, 7.50]	3.90 [2.60, 7.50]	4.00 [2.40, 7.50]

### Full mode

**Figure 2:**
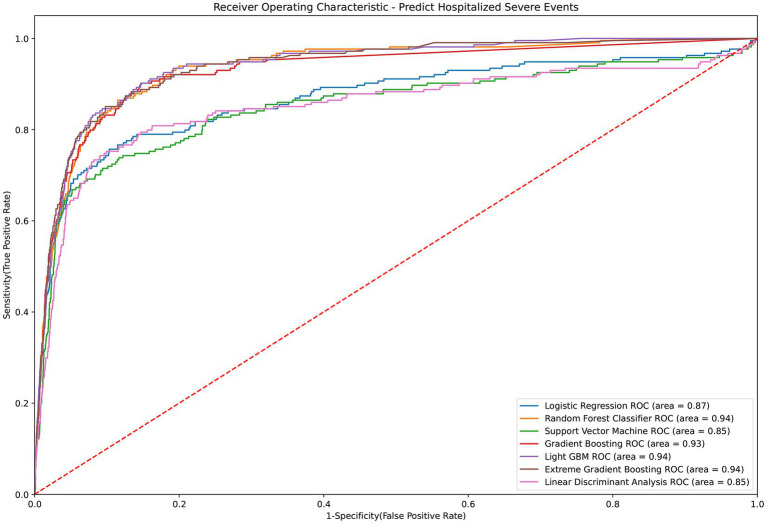
ROC curve of performance of prediction models of severe outcomes or mortality under the full mode.

**Figure 3:**
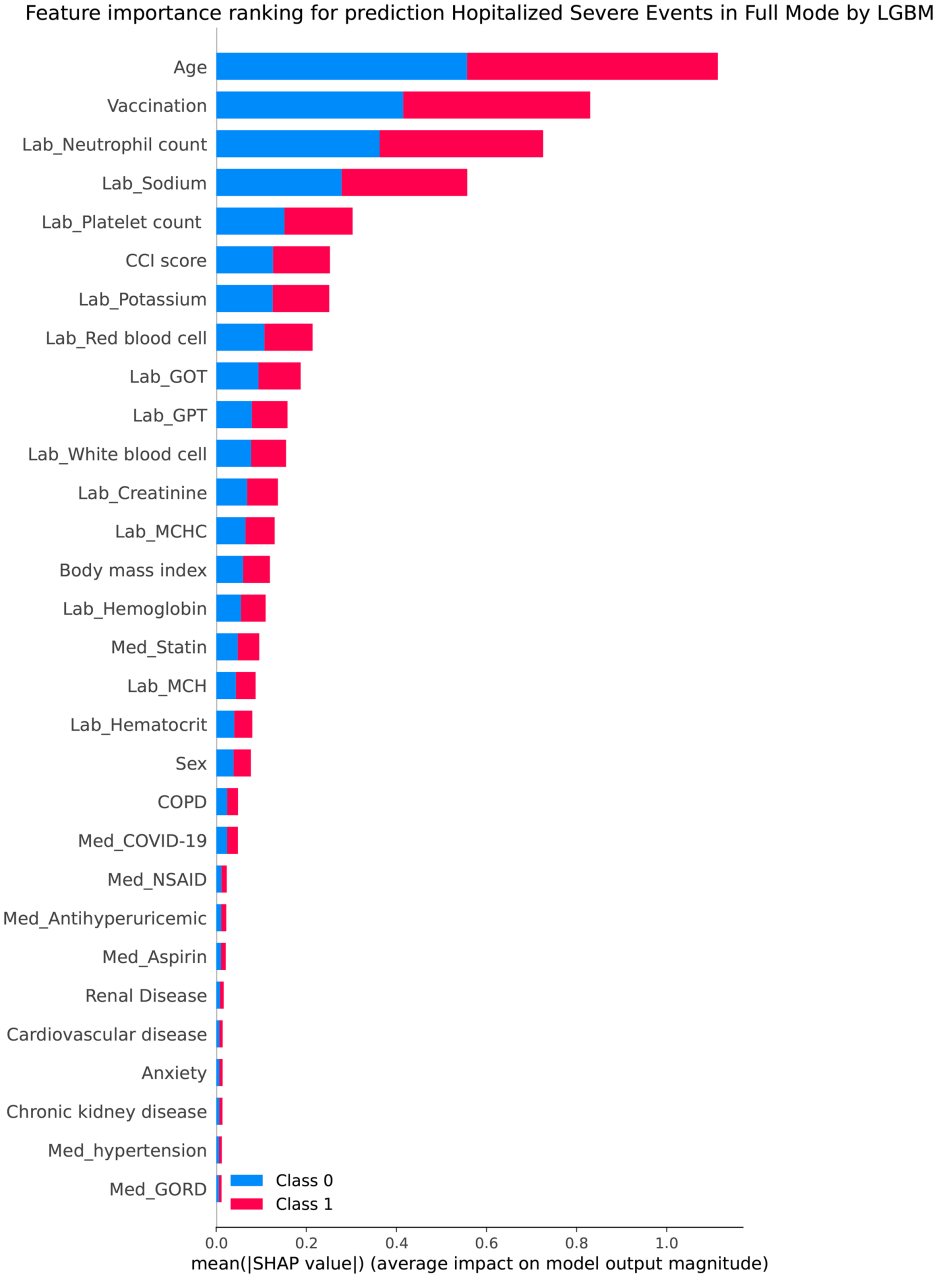
Shapley additive explanations chart of the feature importance for predicting severe outcomes or mortality by the best model under the full mode.

Table 2 presents the performance evaluation of prediction models for overall severe outcome prediction, encompassing mortality, in the full mode. Upon analyzing the test outcomes, the Light GBM model exhibited the highest AUROC (0.939), surpassing other models including XGBoost (AUROC = 0.938), GBM (AUROC = 0.937), RF (AUROC = 0.936), LR (AUROC = 0.869), SVM (AUROC = 0.852), and LDA (AUROC = 0.852). The best-performing model (Light GBM) demonstrated accuracy, sensitivity, and specificity of 85.5%, 0.897, and 0.853, respectively. The cross-validation performance is provided in the Supplementary Appendices 6 and 8. In the cross-validation performance, the Light GBM had the consistent result with the external AUC at 0.924. Figure 2 illustrates the AUROC values of different models in the context of the full mode. The ROC curve delineating the performance of the prediction models for each specific outcome is provided in Supplementary Appendix 3(A). Figure 3 presents the feature importance for predicting severe outcomes or mortality using the optimal model within the full mode. The most significant features were age, vaccination before having PCR test, neutrophil count result, levels of sodium test and platelet count result.

**Table 2:** Performance of prediction models under full mode.

Model	Training AUC	Testing AUC	Accuracy	Sensitivity	Specificity	PPV	NPV	F1-score
Severe outcomes or mortality								
Linear discriminant analysis	0.881	0.852	0.893	0.748	0.901	0.276	0.986	0.524
Logistic regression	0.885	0.869	0.890	0.752	0.897	0.269	0.986	0.572
Support vector machine	0.882	0.852	0.876	0.734	0.884	0.242	0.985	0.532
Random forest	0.949	0.936	0.884	0.860	0.885	0.275	0.992	0.634
Gradient boosting	0.950	0.937	0.859	0.897	0.857	0.242	0.994	0.651
Light GBM	0.991	0.939	0.855	0.897	0.853	0.236	0.994	0.657
Extreme gradient boosting	0.952	0.938	0.899	0.846	0.902	0.304	0.991	0.640
Ventilator use								
Linear discriminant analysis	0.879	0.860	0.883	0.777	0.888	0.248	0.988	0.523
Logistic regression	0.885	0.866	0.845	0.797	0.847	0.199	0.989	0.570
Support vector machine	0.878	0.851	0.887	0.762	0.893	0.254	0.987	0.573
Random forest	0.947	0.938	0.847	0.891	0.845	0.215	0.994	0.630
Gradient boosting machine	0.949	0.937	0.831	0.916	0.827	0.202	0.995	0.631
Light GBM	0.993	0.931	0.884	0.847	0.885	0.260	0.992	0.623
Extreme gradient boosting	0.950	0.938	0.864	0.881	0.863	0.235	0.993	0.654
Intubation use								
Linear discriminant analysis	0.857	0.874	0.884	0.795	0.885	0.058	0.998	0.116
Logistic regression	0.834	0.906	0.850	0.846	0.850	0.048	0.998	0.192
Support vector machine	0.802	0.827	0.894	0.692	0.896	0.056	0.997	0.151
Random forest	0.949	0.880	0.860	0.795	0.861	0.048	0.998	0.161
Gradient boosting machine	0.925	0.872	0.938	0.769	0.939	0.101	0.998	0.201
Light GBM	1.000	0.820	0.791	0.718	0.792	0.030	0.997	0.224
Extreme gradient boosting	0.933	0.894	0.924	0.769	0.925	0.084	0.998	0.283
ICU admission								
Linear discriminant analysis	0.909	0.822	0.925	0.647	0.927	0.033	0.999	0.181
Logistic regression	0.904	0.892	0.942	0.647	0.943	0.042	0.999	0.093
Support vector machine	0.893	0.758	0.972	0.529	0.973	0.071	0.998	0.101
Random forest	0.988	0.849	0.896	0.706	0.896	0.026	0.999	0.119
Gradient boosting machine	0.980	0.730	0.954	0.529	0.955	0.044	0.998	0.056
Light GBM	1.000	0.807	0.829	0.647	0.829	0.014	0.998	0.047
Extreme gradient boosting	0.988	0.864	0.952	0.647	0.953	0.050	0.999	0.154
Mortality								
Linear discriminant analysis	0.940	0.928	0.855	0.902	0.855	0.055	0.999	0.344
Logistic regression	0.962	0.965	0.915	0.878	0.915	0.088	0.999	0.396
Support vector machine	0.965	0.942	0.844	0.927	0.843	0.052	0.999	0.381
Random forest	0.984	0.967	0.927	0.927	0.927	0.106	0.999	0.400
Gradient boosting machine	0.987	0.936	0.880	0.927	0.879	0.067	0.999	0.406
Light GBM	1.000	0.972	0.911	0.951	0.911	0.090	1.000	0.448
Extreme gradient boosting	0.988	0.980	0.942	0.951	0.942	0.133	1.000	0.506

### Simplified mode

**Figure 4:**
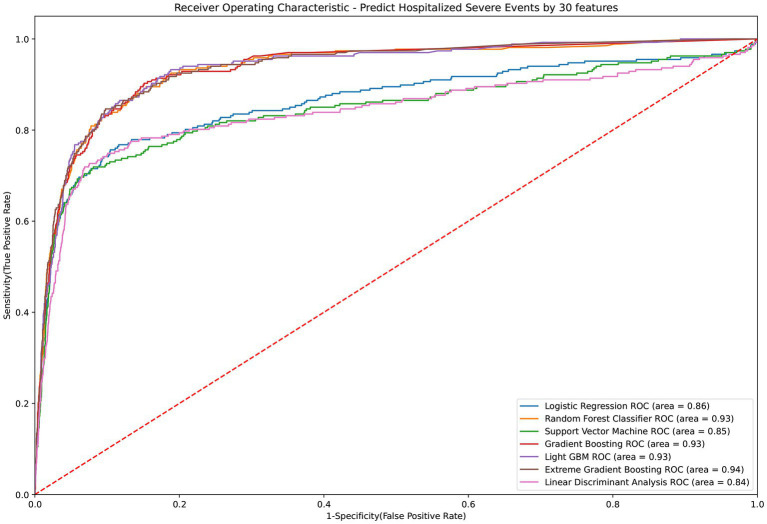
ROC curve of performance of prediction models of severe outcomes or mortality under the simplified mode. ROC, Receiver Operating Characteristic; MED, Medication; Lab, Laboratory result; LGBM, Light Gradient Boosting Machine; CCI, Charlson Comorbidity Index; GOT, Glutamic-oxaloacetic transaminase; GPT, Glutamic-pyruvic transaminase; MCH, Mean corpuscular hemoglobin; MCHC, Mean corpuscular hemoglobin concentration; COPD, Chronic Obstructive Pulmonary Disease; NSAID, Non-steroidal anti-inflammatory drugs; GORD, Gastro-oesophageal reflux disease.

The LGBM algorithm selected the 30 most crucial features from the entire set, which encompassed: sex type, age, BMI, CCI score, vaccination before having PCR test, COVID-19 medications, comorbidities including cardiovascular disease, COPD, renal disease, depression or anxiety, long-term medication such as NSAID, drugs for hypertension, drugs for GORD, aspirin, statin, antihyperuricemic, laboratory test results contain AST (GOT), ALT (GPT), creatinine, RBC, hemoglobin, MCH. MCHC, WBC, Neutrophil, PLT, HCT, NA and K. Table 3 displays the performance evaluation of prediction models for overall severe outcome prediction, inclusive of mortality, in the simplified mode. Based on the results of the tests, the XGBoost model achieved the highest AUROC (0.935) among the other models, namely RF (AUROC = 0.934), Light GBM (AUROC = 0.934), GBM (AUROC = 0.933), LR (AUROC = 0.863), SVM (AUROC = 0.846), and LDA (AUROC = 0.841). The optimal model (XGBoost) achieved accuracy, sensitivity, and specificity of 89.9%, 0.843, and 0.902, respectively. The XGBoost model demonstrates consistent performance when using the cross-validation strategy, with an external AUC of 0.934 The cross-validation performance of the prediction of individual indicators in the simple mode is shown in Supplementary Appendices 7 and 8. Figure 4 illustrates the AUROC values of different models within the context of the simplified mode. The ROC curve delineating the performance of the prediction models for each specific outcome is provided in Supplementary Appendix 3(B).

**Table 3:** Performance of prediction models under simplified mode.

Model	Training AUC	Testing AUC	Accuracy	Sensitivity	Specificity	PPV	NPV	F1-score
Severe outcomes or mortality								
Linear discriminant analysis	0.886	0.841	0.915	0.723	0.925	0.327	0.985	0.523
Logistic regression	0.889	0.863	0.889	0.753	0.896	0.267	0.986	0.569
Support vector machine	0.885	0.846	0.909	0.715	0.919	0.309	0.985	0.534
Random forest	0.952	0.934	0.848	0.891	0.846	0.226	0.994	0.647
Gradient boosting	0.948	0.933	0.870	0.873	0.870	0.253	0.993	0.627
Light GBM	0.995	0.934	0.882	0.861	0.883	0.271	0.992	0.629
Extreme gradient boosting	0.952	0.935	0.899	0.843	0.902	0.304	0.991	0.635
Ventilator use								
Linear discriminant analysis	0.877	0.852	0.890	0.766	0.895	0.258	0.988	0.520
Logistic regression	0.884	0.871	0.901	0.734	0.909	0.277	0.986	0.555
Support vector machine	0.872	0.849	0.881	0.738	0.888	0.238	0.986	0.550
Random forest	0.948	0.940	0.863	0.885	0.862	0.234	0.994	0.642
Gradient boosting machine	0.945	0.939	0.864	0.893	0.863	0.236	0.994	0.637
Light GBM	0.993	0.933	0.905	0.833	0.908	0.302	0.991	0.641
Extreme gradient boosting	0.950	0.940	0.857	0.897	0.855	0.228	0.994	0.653
Intubation use								
Linear discriminant analysis	0.839	0.855	0.833	0.837	0.833	0.043	0.998	0.136
Logistic regression	0.833	0.887	0.879	0.796	0.880	0.056	0.998	0.170
Support vector machine	0.786	0.786	0.846	0.735	0.847	0.041	0.997	0.182
Random forest	0.952	0.892	0.885	0.796	0.885	0.058	0.998	0.171
Gradient boosting machine	0.915	0.875	0.881	0.735	0.882	0.053	0.997	0.154
Light GBM	1.000	0.867	0.902	0.673	0.904	0.059	0.997	0.244
Extreme gradient boosting	0.933	0.902	0.921	0.735	0.923	0.078	0.997	0.314
ICU admission								
Linear discriminant analysis	0.906	0.795	0.941	0.667	0.942	0.042	0.999	0.157
Logistic regression	0.917	0.862	0.963	0.667	0.964	0.066	0.999	0.105
Support vector machine	0.844	0.854	0.762	0.810	0.762	0.013	0.999	0.098
Random forest	0.988	0.886	0.943	0.714	0.944	0.046	0.999	0.127
Gradient boosting machine	0.972	0.872	0.946	0.714	0.947	0.048	0.999	0.130
Light GBM	1.000	0.840	0.773	0.762	0.773	0.013	0.999	0.094
Extreme gradient boosting	0.987	0.880	0.957	0.714	0.958	0.060	0.999	0.208
Mortality								
Linear discriminant analysis	0.936	0.932	0.885	0.902	0.885	0.068	0.999	0.389
Logistic regression	0.962	0.965	0.848	0.961	0.847	0.055	1.000	0.422
Support vector machine	0.966	0.966	0.898	0.922	0.898	0.077	0.999	0.456
Random forest	0.962	0.965	0.848	0.961	0.847	0.055	1.000	0.422
Gradient boosting machine	0.984	0.924	0.871	0.922	0.871	0.062	0.999	0.370
Light GBM	1.000	0.965	0.922	0.922	0.922	0.099	0.999	0.394
Extreme gradient boosting	0.988	0.974	0.916	0.941	0.916	0.094	0.999	0.493

The calibration plot showcasing the performance of prediction models for severe outcomes or mortality can be found in Supplementary Appendix 4. Additionally, the calibration plots illustrating the performance of prediction models for specific outcomes are furnished in Supplementary Appendix 5.

## Discussion

Precise and personalized assessment of individuals at risk of developing severe COVID-19 outcomes holds the potential to enhance both the efficacy of clinical interventions and the judicious utilization of medical resources (17, 18). Several pivotal factors contribute to the heightened predictive capacity of machine learning (ML) models compared to conventional techniques. The considerable advantage of ML models lies in their capacity to generate predictions from vastly expanded datasets, a facet not to be understated. Moreover, ML models remain impervious to human emotions and subjective perspectives, thereby ensuring the objectivity and impartiality of the predictive process. Simultaneously, the innate adaptability inherent to ML models empowers them to swiftly acclimate and assimilate alterations, thereby amplifying their responsiveness to dynamic environments. Ultimately, ML models exhibit an aptitude for discerning intricate patterns of great complexity, often surpassing the capabilities of conventional methodologies. The choice of seven unique machine learning algorithms in this study is based on a comprehensive approach to developing personalized prediction models (19). The algorithms were chosen based on careful evaluation of their attributes and capabilities, ensuring they were in line with the project’s goals and the specific peculiarities of the dataset. The prediction models were developed by employing a range of algorithms, including traditional ones like LDA and LR, as well as basic methods like SVM. Additionally, this study utilize ensemble techniques that involve tree-based algorithms such as RF, GBM, Light GBM, and XGBoost (20, 21).

While prior investigations have constructed and validated predictive models with the goal of forecasting COVID-19 outcomes (22, 23), this study boasts several notable strengths. Firstly, it adeptly harnessed a more diverse and comprehensive dataset than its antecedents, encapsulating demographic particulars, COVID-19 vaccination statuses, COVID-19 drug utilization, comorbidities, long-term medication histories, and results from laboratory tests. Notably, this extends beyond the purview of earlier studies, which omitted the inclusion of long-term medication records and laboratory test outcomes (23–25). Furthermore, distinct from conventional algorithms, this study also employed advanced algorithms, a measure that facilitated the attainment of heightened precision in predictive models. Lastly, through a meticulous analysis of feature significance, this study procured a collection of the most pivotal predictors profoundly impacting model performance (6, 26, 27). The meticulous and personalized appraisal of patients susceptible to severe COVID-19 would undoubtedly amplify the efficacy of clinical interventions and streamline the judicious allocation of medical resources.

This study elucidates that the age of COVID-19 patients stands as the foremost predictor of severe outcome risk, aligning harmoniously with the conclusions drawn from diverse antecedent observational studies, which consistently affirm that elderly COVID-19 patients exhibit a heightened vulnerability to severe outcomes (27–29). Furthermore, this study’s findings expound upon the notion that pre-infection vaccination of COVID-19 patients equally serves as a pivotal predictor of serious events’ risk (including ventilator utilization, intubation, and mortality), as its primary function lies in averting the manifestation of numerous severe outcome risks. This alignment with prior research findings attests to the study’s robustness (30–32).

Presently, numerous national health authorities have issued declarations stipulating the utilization of antiviral agents against COVID-19, notably paxlovid (for individuals aged ≥12 years and weighing ≥40 kg) and molnupiravir (for individuals aged ≥18 years), as a crucial treatment avenue for at-risk patients (33). Zheng et al. conducted a meta-analysis, revealing Paxlovid’s efficacy and safety in managing high-risk COVID-19 patients (34). Debbiny et al.’s outcomes further underscored Paxlovid’s heightened efficacy within vulnerable demographics, encompassing elderly patients, those under immunosuppression, and individuals contending with underlying neurological or cardiovascular conditions (35). Concurrently, Benaicha et al.’s meta-analysis showcased the substantial reduction in all-cause mortality and hospitalization risk attributed to molnupiravir (36). Remarkably, this study’s findings reinforce the pivotal role of COVID-19 antiviral agents in predicting severe outcome risks. Post-COVID-19 infection, individuals incorporating COVID-19 antiviral medications within their treatment regimens evinced a substantial decline in the necessity for ventilator assistance within a three-month timeframe, vis-à-vis counterparts devoid of such treatment. The alignment of the predictive model with antecedent research outcomes underscores its congruence with established clinical practice and the prudent integration of prior findings.

The findings further highlight the significance of prolonged utilization of specific medications (such as benzodiazepines) as a salient affirmative predictor of severe outcome risk, a trend congruent with precedent observational investigations. This discovery bears noteworthy implications within clinical contexts (29). Benzodiazepines, encompassing medications frequently employed to address insomnia, anxiety, seizures, and alcohol withdrawal syndromes, interface with gamma-aminobutyric acid (GABA) receptors within the central nervous system, engendering a tranquilizing and pacifying impact upon the physiological framework. Notably, alongside the potential for immunosuppressive reactions entailing benzodiazepine administration, protracted usage might entail diminished respiratory function, exacerbating complexities among COVID-19 patients (37, 38).

Moreover, study’s investigation unveiled the substantial predictive potency of laboratory test outcomes, encompassing neutrophil count, white blood cell count, platelet count, MCH, and GOT, GPT, NA, and K levels. These variables assumed pivotal roles in the formulation of the predictive model, due to their influential role in disease progression. According to other systematic reviews, high blood White Blood Cell count (WBC), high blood aspartate aminotransferase (AST), high blood C-reactive protein (CRP), low blood platelet count, and a decrease in lymphocyte count may increase the possibilities of severe COVID-19 symptoms (39, 40). Hence, these variables assumed pivotal roles in the formulation of the predictive model, due to their influential role in disease progression.

Nonetheless, this study does encompass certain limitations. Primarily, it hinges upon electronic health records culled from diverse hospitals, constituting the primary wellspring of data. While these records amass a wealth of clinical intricacies, such as demographic particulars, disease management particulars, comprehensive medical histories incorporating comorbidities, prolonged medication use, and pivotal diagnostic outcomes, they regrettably omit several other data categories of import. Absent from this compilation are diverse facets of an individual’s lifestyle, spanning dietary habits, physical activity, tobacco and alcohol consumption, as well as socioeconomic indicators. In prospective endeavors, incorporation of this omitted information might yield alternative predictive models. In clinical practices, hospitals can adopt similar models to assist physicians in the prognostic process. However, a major obstacle is the limited availability and quality of data. The selection of these features was meticulously made, taking into account the available literature. While multiple features were employed in the study, the ones the study utilized are highly accessible and easily obtainable in the electronic health record (EHR) system. Therefore, our findings can be readily applied in future research. The issue of model interpretability is of utmost importance, as healthcare practitioners may struggle to comprehend complex machine learning algorithms. To improve the model’s interpretability, SHAP value ranking was additionally conducted in the findings.

Secondarily, it merits mention that the hospital-held electronic health records solely chronicle the specifics of a patient’s clinical visits, bypassing documentation of medical procedures and interventions executed within other healthcare institutions. Consequently, the clinical insights accessible for each patient might not have attained a truly all-encompassing status, potentially culminating in inaccuracies within the predictions of the predictive model.

Finally, a veritable acknowledgement is that the data origination in this study emanates solely from clinical archives of three hospitals within a singular Taiwanese system. While these hospitals have the largest number of COVID-19 patients in Taiwan, the study may not fully represent the entire population of Taiwan. Therefore, these models, which rely exclusively on hospital cases specific to Northern Taiwan, may have limitations in terms of the generalizability of their findings. Hence, for forthcoming research, it is prudent to foster inter-hospital collaboration and international partnership. Standardized case selection, research blueprinting, data structuring, processing methodologies, and analytical tools—when conjoined with predictive models engendered through multi-center federated learning—will furnish the substratum for the impending research trajectory.

## Conclusion

This study has successfully developed an innovative and precise computer-aided risk prediction model designed to anticipate severe outcomes (including ventilator use, intubation, and intensive care unit admission) or mortality among COVID-19 patients. The outcomes of this research reveal that both the comprehensive and simplified models achieved an area under the curve (AUC) exceeding 0.9, accompanied by an accuracy rate surpassing 85%. The potential to apply timely medical interventions tailored to high-risk patients holds promise for preventing adverse outcomes and thereby ameliorating the disease’s impact on a substantial patient cohort. Although prediction model in this study performed well in the test set, one limitation of this study is the need to take into account the dataset’s representation. The future focus will be on externally validating the model. Collaboration with both domestic hospitals in Taiwan and hospitals in other countries, along with the utilization of the international database, is imperative. There is an expectation that further hospitals in southern Taiwan will be used to validate and enhance this model.

## Data availability statement

The original contributions presented in the study are included in the article/Supplementary material, further inquiries can be directed to the corresponding author.

## Ethics statement

The studies involving humans were approved by the Taipei Medical University–Joint Institutional Review Board (TMU-JIRB no. N202302020). The studies were conducted in accordance with the local legislation and institutional requirements. Written informed consent for participation was not required from the participants or the participants’ legal guardians/next of kin because all data were anonymized and de-identified before the analysis.

## Author contributions

NH: Conceptualization, Formal analysis, Methodology, Writing – original draft, Writing – review & editing, Visualization. F-JT: Writing – review & editing. Y-HC: Conceptualization, Methodology, Writing – original draft, Visualization, Writing – review & editing. WB: Conceptualization, Writing – original draft, Visualization. PP: Data curation, Formal analysis, Methodology, Software, Writing – review & editing. P-AN: Data curation, Formal analysis, Methodology, Visualization, Writing – review & editing. DH: Writing – review & editing. CS-KL: Writing – review & editing. T-CL: Writing – review & editing. C-IC: Writing – review & editing. M-HH: Writing – review & editing. CYL: Writing – review & editing. C-WH: Writing – review & editing. H-CY: Writing – review & editing. JH: Conceptualization, Data curation, Methodology, Project administration, Resources, Supervision, Validation, Writing – review & editing.

## Glossary

**Table:** 

AC	before meals
AI	artificial intelligence
ALT	alanine transaminase, alanine transaminase
AST	aspartate aminotransferase
AUC	area under the curve
AUROC	area under the receiver operating characteristics curve
BMI	body-mass index
BUN	blood urea nitrogen
BUN	blood urea nitrogen
BZD	benzodiazepine
CI	confidence interval
CCI	Charlson comorbidity index
CHF	congestive heart failure
COPD	chronic obstructive pulmonary disease
COVID-19	coronavirus disease 2019
CVA	cerebrovascular disease
CVD	cardiovascular disease
DM	diabetes mellitus
DPP-4i	dipeptidyl peptidase 4 inhibitor
FN	false negative
FP	false positive
GABA	gamma-aminobutyric acid
GBM	Gradient Boosting Machine
GLP-1	glucagon-like peptide-1 analogue
GORD	gastro-oesophageal reflux disease
GOT	glutamic-oxaloacetic transaminase
GPT	glutamic-pyruvic transaminase
HbA1c	glycated hemoglobin
HDL	high-density lipoprotein
HCT	hematocrit
HGB	hemoglobin
HIV/AIDS	human immunodeficiency virus/acquired immune deficiency syndrome
HTN	hypertension
ICD-9-CM	International Classification of Disease, Clinical Modification, Ninth Revision
ICD-10-CM	International Classification of Disease, Clinical Modification, Tenth Revision
ICU	intensive care unit
K	potassium
LDA	linear discriminant analysis
LDL	low-density lipoprotein
LGBM	Light Gradient Boosting Machine
LR	logistic regression
MCH	corpuscular hemoglobin
MCHC	corpuscular hemoglobin concentration
MI	myocardial infarction
MICE	Multiple Imputation by Chained Equations
ML	machine learning
MSSQL	Microsoft Structured Query Language
NA	sodium
NPV	negative predictive value
NSAID	non-steroidal anti-inflammatory drug
OR	odds ratio
PD	Parkinson’s disease
PLT	platelet
PPV	positive predictive value
PUD	peptic ulcer disease
PVD	peripheral vascular disease
RBC	red blood cells
RF	random forest
ROC	receiver operating characteristic
RT-PCR	real-time reverse transcription polymerase chain reaction
S.D.	standard deviation
SGLT2i	sodium-glucose co-transporter 2 inhibitor
SHAP	SHapley Additive exPlanations
SHH	Shuang-Ho Hospital
SVM	Support Vector Machine
SVC	Support Vector Classifier
T1DM	type I diabetes mellitus
T2DM	type II diabetes mellitus
TC	total cholesterol
TG	triglycerides
TMU	Taipei Medical University
TMUCRD	Taipei Medical University Clinical Research Database
TMU-JIRB	Taipei Medical University Joint Institutional Review Board
TMUH	Taipei Medical University Hospital
TN	true negative
TP	true positive
UA	uric acid
WBC	white blood cell
WFH	Wan-Fang Hospital
XGB	eXtreme gradient boosting
