# Supplementary material for: Unveiling the future of COVID-19 patient care: groundbreaking prediction models for severe outcomes or mortality in hospitalized cases
Source: Front Med (Lausanne). 2024 Jan 5;10:1289968. doi: 10.3389/fmed.2023.1289968 (PMC10797111; doi:10.3389/fmed.2023.1289968)
Supplement: Supplementary file 1 [file Data_Sheet_1.PDF]

Appendix

1. Machine learning algorithms used in this study

Linear Discriminant Analysis (LDA)

LDA stands as a prominently utilized dimensionality reduction technique within the realm of machine learning, specifically employed to address classification problems involving more than two classes.(1, 2) Additionally, it is acknowledged as a foundational preprocessing step for capturing disparities in both machine learning modeling and pattern classification applications.(3) For this study, we configured the parameter 'shrinkage' to '0' and designated the solver as 'lsqr' in order to augment the accuracy of estimation and classification.

Logistic Regression (LR)

Logistic regression represents a classification model grounded in the probabilistic framework, which facilitates the estimation of relationships between multiple explanatory variables.(4, 5, 6) Widely employed across diverse fields including biostatistics, clinical medicine, and quantitative psychology, it is characterized by the following equation: where 'x' signifies the input value, 'y' represents the anticipated output, 'b0' corresponds to the bias or intercept term, and 'b1' denotes the coefficient of the input ('x').(5, 7) In the present study, the parameter configurations employed for modeling encompassed a 'class weight' of 0.5 for all categorical variables, 'solver' set to 'liblinear,' 'penalty' set as 'l1,' and 'C' set to 0.1.

$$y = \frac{e^{(b_0 + b_1 X)}}{1 + e^{(b_0 + b_1 X)}}$$

Support Vector Machine (SVM)

The Support Vector Machine (SVM) stands as a supervised machine learning algorithm extensively employed for both classification and regression tasks.(8) The SVM equation can be represented by the following formula, where 'K(x, xi)' signifies the kernel function, 'αI' and 'αi*' denote Lagrange multipliers, and 'b' represents the bias term.(9) In this research, we performed computations employing a linear kernel.

$$f(x) = \sum_{i=1}^{N} (\alpha_i^* - \alpha_i) K(x, x_i) + B$$

Random Forest (RF)

Random Forest constitutes an ensemble learning technique applied to a spectrum of tasks, encompassing classification, regression, and various other objectives.(10) The core principle involves the generation of an extensive array of decision trees during training. The model's output is subsequently determined via voting algorithms, harmonizing the outcomes from each individual tree. More specifically, the output is synthesized through the application of voting algorithms, which amalgamate the outputs from each constituent tree.(10, 11) Within this investigation, we advocate the establishment of the ensuing parameter configurations: 'n_estimators' (number of trees) set to 500, 'max_depth' limited to 10, 'min_samples_split' fixed at 400, and 'class_weight' equated to 0.5 for each class.

Gradient Boosting Machine (GBM)

Gradient Boosting Machine (GBM) is a technique distinguished for its remarkable predictive speed and accuracy, particularly in managing extensive and intricate datasets.(12) The

inherent capacity of TreeBoost procedures to swiftly gauge potential predictive performance, coupled with their exceptional resilience, renders them a valuable preprocessing instrument suited for handling imperfect data.(13) In this study, the GBM model was implemented with the following parameter settings: 'learning_rate' set to 0.1, 'max_depth' limited to 3, 'n_estimators' configured at 50, and 'subsample' defined as 0.7."

Light Gradient Boosting machine (LGBM)

LGBM represents a gradient boosting framework founded upon decision trees, engineered to enhance model efficiency and optimize memory utilization. Employing a histogram-based technique, LGBM entails categorizing data into bins guided by the histogram distribution.(14) For this project, the LGBM model was harnessed with its default settings, encompassing default parameters.

eXtreme Gradient Boosting (XGBoost)

XGBoost stands as an open-source software library, leveraging the Gradient Boosting framework to enact efficient distributed gradient boosting machine learning algorithms. As a gradient-boosted decision tree (GBDT) machine learning library, XGBoost exhibits scalability and distribution capabilities. It boasts parallel tree boosting functionality and holds its position as the preeminent machine learning library for addressing regression, classification, and ranking challenges.(15) Within this research endeavor, the XGBoost model was instantiated with the subsequent configuration parameters: 'learning_rate' set to 0.1, 'max_depth' constrained to 3, 'n_estimators' established at 50, and 'subsample' stipulated as 0.7.

2. Baseline table of patient characteristics

Variables	Total (N = 22192)	Ventilator (N=1010)	No ventilator (N=21182)	Intubation (N=196)	No intubation (N=21996)	ICU (N=85)	No ICU (N=22107)	Mortality (N = 205)	No mortality (N = 21987)
**Demographic information**									
**Gender, N (%)**									
Female	12452 (56.1%)	459 (45.4%)	11993 (56.6%)	84 (42.9%)	12368 (56.2%)	28 (32.9%)	12424 (56.2%)	72 (35.1%)	12380 (56.3%)
Male	9740 (43.9%)	551 (54.6%)	9189 (43.4%)	112 (57.1%)	9628 (43.8%)	57 (67.1%)	9683 (43.8%)	133 (64.9%)	9607 (43.7%)
**Age, N (%)**									
Mean (SD)	49.3 (17.4)	71.4 (17.2)	48.2 (16.7)	66.5 (15.0)	49.1 (17.3)	72.9 (13.6)	49.2 (17.4)	78.2 (12.4)	49.0 (17.2)
Median [Min, Max]	47.4 [20.0, 110]	73.5 [20.0, 108]	46.4 [20.0, 110]	69.4 [20.0, 97.7]	47.2 [20.0, 110]	72.8 [32.2, 97.7]	47.3 [20.0, 110]	79.5 [43.1, 102]	47.2 [20.0, 110]
Age < 65 yrs.	17625 (79.4%)	303 (30.0%)	17322 (81.8%)	77 (39.3%)	17548 (79.8%)	17 (20.0%)	17608 (79.6%)	28 (13.7%)	17597 (80.0%)
65 ≤ Age < 85 yrs.	3960 (17.8%)	459 (45.4%)	3501 (16.5%)	100 (51.0%)	3860 (17.5%)	52 (61.2%)	3908 (17.7%)	110 (53.7%)	3850 (17.5%)
Age ≥85 yrs.	607 (2.7%)	248 (24.6%)	359 (1.7%)	19 (9.7%)	588 (2.7%)	16 (18.8%)	591 (2.7%)	67 (32.7%)	540 (2.5%)
**Health status**									
**BMI, N (%)**									
Mean (SD)	24.4 (4.51)	23.8 (4.71)	24.4 (4.49)	25.1 (4.83)	24.3 (4.50)	24.8 (4.49)	24.4 (4.51)	23.1 (4.39)	24.4 (4.51)
Median [Min, Max]	23.8 [9.21, 51.9]	23.4 [12.5, 48.5]	23.9 [9.21, 51.9]	24.4 [15.7, 43.8]	23.8 [9.21, 51.9]	24.0 [16.9, 37.8]	23.8 [9.21, 51.9]	22.4 [13.5, 41.6]	23.8 [9.21, 51.9]
BMI < 18.5	730 (3.3%)	95 (9.4%)	635 (3.0%)	14 (7.1%)	716 (3.3%)	4 (4.7%)	726 (3.3%)	21 (10.2%)	709 (3.2%)
18.5 <= BMI < 24	5314 (23.9%)	397 (39.3%)	4917 (23.2%)	63 (32.1%)	5251 (23.9%)	36 (42.4%)	5278 (23.9%)	96 (46.8%)	5218 (23.7%)
BMI >= 24	5651 (25.5%)	401 (39.7%)	5250 (24.8%)	96 (49.0%)	5555 (25.3%)	40 (47.1%)	5611 (25.4%)	68 (33.2%)	5583 (25.4%)
**CCI score, N (%)**									
Mean (SD)	0.530 (1.52)	1.88 (2.68)	0.465 (1.41)	1.76 (2.41)	0.519 (1.51)	1.89 (2.84)	0.524 (1.51)	1.80 (3.08)	0.518 (1.50)
Median [Min, Max]	0 [0, 18.0]	0 [0, 16.0]	0 [0, 18.0]	1.00 [0, 11.0]	0 [0, 18.0]	0 [0, 16.0]	0 [0, 18.0]	0 [0, 16.0]	0 [0, 18.0]
CCI score = 0	18298 (82.5%)	517 (51.2%)	17781 (83.9%)	95 (48.5%)	18203 (82.8%)	44 (51.8%)	18254 (82.6%)	131 (63.9%)	18167 (82.6%)
0 <= CCI score < 3	2115 (9.5%)	187 (18.5%)	1928 (9.1%)	44 (22.4%)	2071 (9.4%)	16 (18.8%)	2099 (9.5%)	17 (8.3%)	2098 (9.5%)
CCI score >= 3	1779 (8.0%)	306 (30.3%)	1473 (7.0%)	57 (29.1%)	1722 (7.8%)	25 (29.4%)	1754 (7.9%)	57 (27.8%)	1722 (7.8%)
**COVID-19-related details**									
COVID-19 vaccine	5820 (26.2%)	151 (15.0%)	5669 (26.8%)	23 (11.7%)	5797 (26.4%)	9 (10.6%)	5820 (26.2%)	24 (11.7%)	5796 (26.4%)
Covid-19 medications (Paxlovid or Molnupiravir)	558 (2.5%)	49 (4.9%)	509 (2.4%)	4 (2.0%)	554 (2.5%)	2 (2.4%)	558 (2.5%)	4 (2.0%)	554 (2.5%)
**Comorbidities, N (%)**									

Myocardial infarction (MI)	126 (0.6%)	30 (3.0%)	96 (0.5%)	7 (3.6%)	119 (0.5%)	2 (2.4%)	124 (0.6%)	3 (1.5%)	123 (0.6%)
Congestive heart failure (CHF)	534 (2.4%)	111 (11.0%)	423 (2.0%)	17 (8.7%)	517 (2.4%)	5 (5.9%)	529 (2.4%)	17 (8.3%)	517 (2.4%)
Peripheral vascular disease	161 (0.7%)	19 (1.9%)	142 (0.7%)	3 (1.5%)	158 (0.7%)	1 (1.2%)	160 (0.7%)	2 (1.0%)	159 (0.7%)
Cardiovascular disease	997 (4.5%)	182 (18.0%)	815 (3.8%)	34 (17.3%)	963 (4.4%)	17 (20.0%)	980 (4.4%)	29 (14.1%)	968 (4.4%)
Dementia	285 (1.3%)	95 (9.4%)	190 (0.9%)	9 (4.6%)	276 (1.3%)	3 (3.5%)	282 (1.3%)	19 (9.3%)	266 (1.2%)
COPD	1106 (5.0%)	155 (15.3%)	951 (4.5%)	30 (15.3%)	1076 (4.9%)	13 (15.3%)	1093 (4.9%)	23 (11.2%)	1083 (4.9%)
Rheumatic disease	160 (0.7%)	18 (1.8%)	142 (0.7%)	2 (1.0%)	158 (0.7%)	1 (1.2%)	159 (0.7%)	1 (0.5%)	159 (0.7%)
Peptic ulcer disease	1367 (6.2%)	153 (15.1%)	1214 (5.7%)	30 (15.3%)	1337 (6.1%)	14 (16.5%)	1353 (6.1%)	26 (12.7%)	1341 (6.1%)
Liver disease	860 (3.9%)	85 (8.4%)	775 (3.7%)	22 (11.2%)	838 (3.8%)	9 (10.6%)	851 (3.8%)	22 (10.7%)	838 (3.8%)
Diabetes mellitus	1347 (6.1%)	210 (20.8%)	1137 (5.4%)	31 (15.8%)	1316 (6.0%)	17 (20.0%)	1330 (6.0%)	41 (20.0%)	1306 (5.9%)
Hemiplegia	29 (0.1%)	6 (0.6%)	23 (0.1%)	1 (0.5%)	28 (0.1%)	0 (0%)	29 (0.1%)	3 (1.5%)	26 (0.1%)
Renal disease	673 (3.0%)	131 (13.0%)	542 (2.6%)	26 (13.3%)	647 (2.9%)	13 (15.3%)	660 (3.0%)	29 (14.1%)	644 (2.9%)
Cancer	535 (2.4%)	90 (8.9%)	445 (2.1%)	20 (10.2%)	515 (2.3%)	9 (10.6%)	526 (2.4%)	18 (8.8%)	517 (2.4%)
AIDS/HIV	85 (0.4%)	18 (1.8%)	67 (0.3%)	4 (2.0%)	81 (0.4%)	2 (2.4%)	83 (0.4%)	3 (1.5%)	82 (0.4%)
Hypertension	1490 (6.7%)	240 (23.8%)	1250 (5.9%)	50 (25.5%)	1440 (6.5%)	25 (29.4%)	1465 (6.6%)	45 (22.0%)	1445 (6.6%)
Hyperlipidemia	2055 (9.3%)	213 (21.1%)	1842 (8.7%)	52 (26.5%)	2003 (9.1%)	23 (27.1%)	2032 (9.2%)	31 (15.1%)	2024 (9.2%)
Hyperuricemia	80 (0.4%)	14 (1.4%)	66 (0.3%)	3 (1.5%)	77 (0.4%)	1 (1.2%)	79 (0.4%)	2 (1.0%)	78 (0.4%)
Depression or anxiety	884 (4.0%)	87 (8.6%)	797 (3.8%)	18 (9.2%)	866 (3.9%)	6 (7.1%)	878 (4.0%)	9 (4.4%)	884 (4.0%)
Anemia	621 (2.8%)	92 (9.1%)	529 (2.5%)	21 (10.7%)	600 (2.7%)	7 (8.2%)	614 (2.8%)	17 (8.3%)	604 (2.7%)
Parkinson's disease	136 (0.6%)	34 (3.4%)	102 (0.5%)	4 (2.0%)	132 (0.6%)	1 (1.2%)	135 (0.6%)	6 (2.9%)	130 (0.6%)
Osteoporosis	364 (1.6%)	62 (6.1%)	302 (1.4%)	12 (6.1%)	352 (1.6%)	7 (8.2%)	357 (1.6%)	12 (5.9%)	352 (1.6%)
**Long-term medication records, N (%)**									
BZD	1695 (7.6%)	216 (21.4%)	1479 (7.0%)	42 (21.4%)	1653 (7.5%)	22 (25.9%)	1695 (7.6%)	71 (34.6%)	1624 (7.4%)
NSAID	1016 (4.6%)	65 (6.4%)	951 (4.5%)	20 (10.2%)	996 (4.5%)	10 (11.8%)	1016 (4.6%)	16 (7.8%)	1000 (4.5%)
Aspirin	1396 (6.3%)	178 (17.6%)	1218 (5.8%)	40 (20.4%)	1356 (6.2%)	22 (25.9%)	1396 (6.3%)	54 (26.3%)	1342 (6.1%)
HTN	2846 (12.8%)	323 (32.0%)	2523 (11.9%)	62 (31.6%)	2784 (12.7%)	32 (37.6%)	2846 (12.8%)	90 (43.9%)	2756 (12.5%)
DM	1250 (5.6%)	154 (15.2%)	1096 (5.2%)	25 (12.8%)	1225 (5.6%)	14 (16.5%)	1250 (5.6%)	49 (23.9%)	1201 (5.5%)
Statin	2141 (9.6%)	181 (17.9%)	1960 (9.3%)	43 (21.9%)	2098 (9.5%)	21 (24.7%)	2141 (9.6%)	46 (22.4%)	2095 (9.5%)
Antihyperuricemic	418 (1.9%)	56 (5.5%)	362 (1.7%)	11 (5.6%)	407 (1.9%)	5 (5.9%)	418 (1.9%)	27 (13.2%)	391 (1.8%)
Antihistamin	528 (2.4%)	49 (4.9%)	479 (2.3%)	7 (3.6%)	521 (2.4%)	4 (4.7%)	528 (2.4%)	14 (6.8%)	514 (2.3%)
GORD	1317 (5.9%)	182 (18.0%)	1135 (5.4%)	35 (17.9%)	1282 (5.8%)	21 (24.7%)	1317 (5.9%)	54 (26.3%)	1263 (5.7%)
Steroids	2420 (10.9%)	228 (22.6%)	2192 (10.3%)	56 (28.6%)	2364 (10.7%)	28 (32.9%)	2420 (10.9%)	69 (33.7%)	2351 (10.7%)

Laboratory test results, N (%)									
**HbA1C**									
Mean (SD)	6.31 (1.24)	6.56 (1.56)	6.28 (1.19)	6.48 (1.43)	6.30 (1.23)	6.34 (1.06)	6.31 (1.24)	6.59 (1.75)	6.30 (1.22)
Median [Min, Max]	6.00 [3.90, 16.4]	6.10 [4.10, 16.4]	5.90 [3.90, 16.2]	6.10 [4.50, 12.1]	6.00 [3.90, 16.4]	6.30 [4.50, 9.30]	6.00 [3.90, 16.4]	6.20 [4.80, 16.4]	6.00 [3.90, 16.2]
Missing	18571 (83.7%)	621 (61.5%)	17950 (84.7%)	113 (57.7%)	18458 (83.9%)	49 (57.6%)	18522 (83.8%)	103 (50.2%)	18468 (84.0%)
**TC**									
Mean (SD)	171 (40.4)	158 (44.4)	173 (39.7)	163 (33.8)	172 (40.5)	159 (36.5)	171 (40.4)	152 (57.5)	172 (39.8)
Median [Min, Max]	168 [35.0, 545]	153 [36.0, 545]	169 [35.0, 443]	165 [66.0, 224]	168 [35.0, 545]	162 [82.0, 246]	168 [35.0, 545]	148 [60.0, 545]	168 [35.0, 443]
Missing	18199 (82.0%)	637 (63.1%)	17562 (82.9%)	126 (64.3%)	18073 (82.2%)	54 (63.5%)	18145 (82.1%)	115 (56.1%)	18084 (82.2%)
**HDL**									
Mean (SD)	51.6 (15.4)	46.6 (14.8)	52.1 (15.4)	45.7 (15.4)	51.7 (15.4)	43.6 (18.9)	51.7 (15.4)	41.3 (14.9)	51.8 (15.4)
Median [Min, Max]	50.0 [0, 124]	45.0 [0, 95.0]	50.0 [7.00, 124]	45.0 [0, 95.0]	50.0 [7.00, 124]	40.0 [19.0, 95.0]	50.0 [0, 124]	39.0 [0, 87.0]	50.0 [7.00, 124]
Missing	19203 (86.5%)	758 (75.0%)	18445 (87.1%)	143 (73.0%)	19060 (86.7%)	59 (69.4%)	19144 (86.6%)	140 (68.3%)	19063 (86.7%)
**LDL**									
Mean (SD)	100 (33.4)	90.6 (36.0)	101 (33.0)	90.6 (28.3)	101 (33.5)	87.0 (26.2)	100 (33.5)	87.6 (44.0)	101 (33.1)
Median [Min, Max]	96.0 [16.0, 433]	86.0 [16.0, 433]	97.0 [20.0, 345]	90.0 [42.0, 161]	96.0 [16.0, 433]	90.5 [41.0, 145]	96.0 [16.0, 433]	83.0 [31.0, 433]	97.0 [16.0, 345]
Missing	17930 (80.8%)	622 (61.6%)	17308 (81.7%)	120 (61.2%)	17810 (81.0%)	45 (52.9%)	17885 (80.9%)	109 (53.2%)	17821 (81.1%)
**TG**									
Mean (SD)	123 (80.7)	124 (82.3)	123 (80.6)	121 (75.3)	123 (80.8)	118 (59.7)	123 (80.9)	117 (79.9)	123 (80.7)
Median [Min, Max]	103 [21.0, 1920]	100 [21.0, 656]	103 [26.0, 1920]	99.0 [21.0, 557]	103 [26.0, 1920]	115 [28.0, 307]	103 [21.0, 1920]	98.0 [28.0, 592]	103 [21.0, 1920]
Missing	17817 (80.3%)	609 (60.3%)	17208 (81.2%)	123 (62.8%)	17694 (80.4%)	49 (57.6%)	17768 (80.4%)	108 (52.7%)	17709 (80.5%)
**Uric acid (UA)**									
Mean (SD)	5.75 (1.72)	5.98 (2.12)	5.72 (1.67)	5.94 (1.87)	5.74 (1.72)	5.84 (2.23)	5.75 (1.71)	6.15 (2.79)	5.74 (1.68)
Median [Min, Max]	5.60 [0, 16.9]	6.00 [0, 16.9]	5.60 [0, 14.8]	5.90 [1.30, 11.5]	5.60 [0, 16.9]	6.10 [2.10, 11.5]	5.60 [0, 16.9]	6.00 [1.50, 16.9]	5.60 [0, 14.8]
Missing	19016 (85.7%)	690 (68.3%)	18326 (86.5%)	137 (69.9%)	18879 (85.8%)	56 (65.9%)	18960 (85.8%)	114 (55.6%)	18902 (86.0%)
**AST (GOT)**									
Mean (SD)	30.2 (123)	53.4 (288)	25.5 (35.1)	115 (663)	27.6 (44.6)	53.6 (84.3)	29.8 (123)	113 (597)	26.9 (39.4)
Median [Min, Max]	21.0 [0, 7930]	26.5 [8.00, 7930]	20.0 [0, 1550]	31.0 [11.0, 7930]	21.0 [0, 1680]	32.0 [14.0, 553]	21.0 [0, 7930]	35.0 [8.00, 7930]	21.0 [0, 1680]
Missing	17209 (77.5%)	170 (16.8%)	17039 (80.4%)	48 (24.5%)	17161 (78.0%)	13 (15.3%)	17196 (77.8%)	17 (8.3%)	17192 (78.2%)
**ALT (GPT)**									
Mean (SD)	26.1 (57.1)	37.2 (150)	24.7 (28.4)	57.2 (243)	25.4 (44.9)	37.2 (83.0)	26.0 (56.7)	63.1 (233)	25.1 (43.4)
Median [Min, Max]	19.0 [0, 2690]	18.0 [0, 2690]	19.0 [0, 843]	20.0 [0, 2580]	19.0 [0, 2690]	20.0 [0, 654]	19.0 [0, 2690]	20.0 [0, 2580]	19.0 [0, 2690]

Missing	15810 (71.2%)	297 (29.4%)	15513 (73.2%)	59 (30.1%)	15751 (71.6%)	20 (23.5%)	15790 (71.4%)	41 (20.0%)	15769 (71.7%)
**Total Protein**									
Mean (SD)	17.2 (55.1)	9.48 (14.9)	19.9 (63.4)	9.57 (15.4)	17.5 (56.3)	6.16 (0.937)	17.4 (55.8)	8.47 (12.3)	17.9 (57.3)
Median [Min, Max]	6.90 [0, 807]	6.80 [3.30, 102]	7.00 [0, 807]	6.55 [4.60, 78.6]	6.90 [0, 807]	6.35 [4.50, 7.50]	6.90 [0, 807]	5.95 [3.60, 78.6]	7.00 [0, 807]
Missing	21708 (97.8%)	882 (87.3%)	20826 (98.3%)	174 (88.8%)	21534 (97.9%)	73 (85.9%)	21635 (97.9%)	167 (81.5%)	21541 (98.0%)
**Albumin**									
Mean (SD)	3.89 (0.648)	3.58 (0.622)	3.99 (0.623)	3.57 (0.550)	3.90 (0.648)	3.24 (0.656)	3.90 (0.640)	3.12 (0.651)	3.95 (0.607)
Median [Min, Max]	4.00 [1.50, 5.50]	3.60 [1.70, 5.10]	4.10 [1.50, 5.50]	3.50 [2.50, 4.60]	4.00 [1.50, 5.50]	3.30 [1.50, 4.50]	4.00 [1.60, 5.50]	3.10 [1.50, 4.70]	4.00 [1.80, 5.50]
Missing	20299 (91.5%)	526 (52.1%)	19773 (93.3%)	115 (58.7%)	20184 (91.8%)	44 (51.8%)	20255 (91.6%)	65 (31.7%)	20234 (92.0%)
**Globubin**									
Mean (SD)	2.85 (0.532)	2.99 (0.839)	2.82 (0.426)	3.35 (1.48)	2.84 (0.513)	4.03 (1.46)	2.82 (0.460)	3.42 (1.43)	2.82 (0.428)
Median [Min, Max]	2.70 [1.90, 5.40]	2.90 [2.00, 5.40]	2.70 [1.90, 4.00]	3.35 [2.30, 4.40]	2.70 [1.90, 5.40]	4.20 [2.50, 5.40]	2.70 [1.90, 4.40]	3.25 [2.00, 5.40]	2.70 [1.90, 4.00]
Missing	22079 (99.5%)	988 (97.8%)	21091 (99.6%)	194 (99.0%)	21885 (99.5%)	82 (96.5%)	21997 (99.5%)	199 (97.1%)	21880 (99.5%)
**BUN**									
Mean (SD)	21.6 (19.2)	28.9 (26.7)	20.0 (16.7)	32.5 (37.9)	21.2 (18.2)	36.5 (28.3)	21.4 (18.9)	42.1 (35.7)	20.7 (17.6)
Median [Min, Max]	15.0 [2.00, 287]	19.0 [2.00, 287]	15.0 [3.00, 159]	19.0 [2.00, 287]	15.0 [2.00, 159]	24.5 [6.00, 156]	15.0 [2.00, 287]	31.0 [2.00, 287]	15.0 [2.00, 186]
Missing	17938 (80.8%)	238 (23.6%)	17700 (83.6%)	64 (32.7%)	17874 (81.3%)	19 (22.4%)	17919 (81.1%)	29 (14.1%)	17909 (81.5%)
**Creatinine**									
Mean (SD)	1.28 (1.88)	1.87 (2.51)	1.19 (1.76)	2.00 (2.45)	1.26 (1.86)	2.28 (2.62)	1.27 (1.87)	2.16 (2.37)	1.25 (1.86)
Median [Min, Max]	0.820 [0, 23.3]	1.00 [0, 19.3]	0.800 [0, 23.3]	1.08 [0.340, 17.8]	0.820 [0, 23.3]	1.29 [0.340, 17.8]	0.820 [0, 23.3]	1.27 [0, 17.8]	0.810 [0, 23.3]
Missing	14678 (66.1%)	68 (6.7%)	14610 (69.0%)	18 (9.2%)	14660 (66.6%)	3 (3.5%)	14675 (66.4%)	7 (3.4%)	14671 (66.7%)
**RBC**									
Mean (SD)	4.39 (0.741)	4.08 (0.860)	4.44 (0.704)	4.21 (0.968)	4.39 (0.733)	3.95 (0.812)	4.40 (0.738)	3.74 (0.912)	4.41 (0.725)
Median [Min, Max]	4.44 [1.03, 7.67]	4.11 [1.03, 6.87]	4.47 [1.41, 7.67]	4.34 [1.67, 6.85]	4.44 [1.03, 7.67]	4.02 [2.20, 6.24]	4.44 [1.03, 7.67]	3.75 [1.77, 7.19]	4.45 [1.03, 7.67]
Missing	16058 (72.4%)	78 (7.7%)	15980 (75.4%)	25 (12.8%)	16033 (72.9%)	5 (5.9%)	16053 (72.6%)	8 (3.9%)	16050 (73.0%)
**Hemoglobin (HGB)**									
Mean (SD)	13.0 (2.04)	12.1 (2.39)	13.1 (1.93)	12.4 (2.58)	13.0 (2.02)	12.0 (2.39)	13.0 (2.03)	11.3 (2.61)	13.1 (1.99)
Median [Min, Max]	13.3 [3.40, 25.2]	12.4 [4.50, 18.3]	13.4 [3.40, 25.2]	12.8 [4.70, 17.6]	13.3 [3.40, 25.2]	12.0 [7.00, 16.8]	13.3 [3.40, 25.2]	11.4 [5.80, 16.8]	13.3 [3.40, 25.2]
Missing	15728 (70.9%)	69 (6.8%)	15659 (73.9%)	23 (11.7%)	15705 (71.4%)	4 (4.7%)	15724 (71.1%)	7 (3.4%)	15721 (71.5%)
**MCH**									
Mean (SD)	29.7 (3.11)	29.9 (3.24)	29.7 (3.08)	29.8 (3.59)	29.7 (3.09)	30.6 (3.06)	29.7 (3.11)	30.3 (3.04)	29.7 (3.11)
Median [Min, Max]	30.3 [12.9, 43.4]	30.5 [13.2, 43.4]	30.2 [12.9, 41.9]	30.5 [17.2, 36.7]	30.3 [12.9, 43.4]	30.8 [20.9, 35.3]	30.3 [12.9, 43.4]	30.5 [18.8, 38.4]	30.3 [12.9, 43.4]
Missing	16165 (72.8%)	79 (7.8%)	16086 (75.9%)	26 (13.3%)	16139 (73.4%)	5 (5.9%)	16160 (73.1%)	8 (3.9%)	16157 (73.5%)

**MCHC**									
Mean (SD)	33.8 (1.20)	33.8 (1.44)	33.8 (1.16)	33.6 (1.62)	33.8 (1.19)	33.8 (1.23)	33.8 (1.20)	33.6 (1.33)	33.8 (1.20)
Median [Min, Max]	33.9 [16.6, 40.1]	33.9 [16.6, 40.1]	33.9 [26.1, 37.9]	33.8 [27.2, 40.1]	33.9 [16.6, 37.9]	33.9 [29.7, 36.5]	33.9 [16.6, 40.1]	33.8 [29.6, 37.1]	33.9 [16.6, 40.1]
Missing	16165 (72.8%)	79 (7.8%)	16086 (75.9%)	26 (13.3%)	16139 (73.4%)	5 (5.9%)	16160 (73.1%)	8 (3.9%)	16157 (73.5%)
**WBC**									
Mean (SD)	7.39 (3.31)	8.14 (4.78)	7.26 (2.96)	9.13 (7.30)	7.35 (3.11)	9.59 (6.30)	7.37 (3.24)	10.3 (8.32)	7.30 (2.97)
Median [Min, Max]	6.78 [0.200, 78.7]	7.19 [0.570, 78.7]	6.72 [0.200, 53.2]	7.72 [0.570, 78.7]	6.76 [0.200, 53.2]	8.40 [0.570, 37.8]	6.77 [0.200, 78.7]	8.58 [0.570, 78.7]	6.75 [0.200, 49.2]
Missing	15907 (71.7%)	74 (7.3%)	15833 (74.7%)	24 (12.2%)	15883 (72.2%)	4 (4.7%)	15903 (71.9%)	7 (3.4%)	15900 (72.3%)
**Neutrophil**									
Mean (SD)	67.4 (13.6)	74.3 (13.4)	65.9 (13.1)	74.5 (14.7)	67.2 (13.5)	77.2 (12.5)	67.3 (13.6)	78.8 (13.9)	67.0 (13.4)
Median [Min, Max]	67.4 [0, 99.0]	75.7 [0, 99.0]	65.7 [0, 98.0]	76.5 [0, 96.5]	67.1 [0, 99.0]	78.2 [34.3, 96.5]	67.3 [0, 99.0]	81.5 [0, 98.5]	66.9 [0, 99.0]
Missing	17219 (77.6%)	96 (9.5%)	17123 (80.8%)	32 (16.3%)	17187 (78.1%)	4 (4.7%)	17215 (77.9%)	8 (3.9%)	17211 (78.3%)
**Lymphocyte**									
Mean (SD)	21.6 (11.7)	14.8 (10.2)	23.1 (11.4)	16.1 (12.1)	21.8 (11.6)	13.8 (10.2)	21.8 (11.6)	11.4 (8.72)	22.1 (11.6)
Median [Min, Max]	20.8 [0, 84.0]	12.8 [0, 73.8]	22.9 [0, 84.0]	13.9 [0.500, 73.8]	21.1 [0, 84.0]	11.4 [0.500, 56.7]	21.0 [0, 84.0]	10.2 [0, 56.7]	21.5 [0, 84.0]
Missing	18165 (81.9%)	307 (30.4%)	17858 (84.3%)	56 (28.6%)	18109 (82.3%)	15 (17.6%)	18150 (82.1%)	23 (11.2%)	18142 (82.5%)
**PLT**									
Mean (SD)	230 (80.9)	197 (87.1)	236 (78.3)	190 (88.1)	232 (80.4)	175 (97.6)	231 (80.4)	178 (88.5)	232 (80.1)
Median [Min, Max]	226 [0, 1010]	182 [12.0, 652]	230 [0, 1010]	172 [14.0, 569]	226 [0, 1010]	151 [14.0, 569]	226 [0, 1010]	155 [14.0, 478]	227 [0, 1010]
Missing	16033 (72.2%)	75 (7.4%)	15958 (75.3%)	25 (12.8%)	16008 (72.8%)	4 (4.7%)	16029 (72.5%)	7 (3.4%)	16026 (72.9%)
**HCT**									
Mean (SD)	38.3 (5.85)	35.8 (6.91)	38.8 (5.52)	36.8 (7.44)	38.4 (5.79)	35.5 (6.85)	38.4 (5.83)	33.4 (7.68)	38.5 (5.71)
Median [Min, Max]	39.1 [10.4, 55.5]	36.8 [11.7, 52.0]	39.4 [10.4, 55.5]	37.9 [11.7, 51.3]	39.1 [10.4, 55.5]	35.8 [22.0, 48.4]	39.1 [10.4, 55.5]	33.4 [16.4, 50.6]	39.2 [10.4, 55.5]
Missing	16016 (72.2%)	77 (7.6%)	15939 (75.2%)	25 (12.8%)	15991 (72.7%)	5 (5.9%)	16011 (72.4%)	8 (3.9%)	16008 (72.8%)
**NA**									
Mean (SD)	138 (4.52)	135 (6.01)	138 (3.93)	136 (6.68)	138 (4.41)	135 (5.48)	138 (4.49)	137 (7.28)	138 (4.37)
Median [Min, Max]	138 [68.5, 167]	136 [103, 167]	139 [68.5, 162]	136 [103, 163]	138 [68.5, 167]	135 [111, 146]	138 [68.5, 167]	137 [111, 162]	138 [68.5, 167]
Missing	17111 (77.1%)	90 (8.9%)	17021 (80.4%)	33 (16.8%)	17078 (77.6%)	6 (7.1%)	17105 (77.4%)	8 (3.9%)	17103 (77.8%)
**K**									
Mean (SD)	4.04 (0.538)	3.98 (0.661)	4.05 (0.508)	4.05 (0.762)	4.04 (0.529)	4.13 (0.866)	4.03 (0.531)	4.11 (0.781)	4.03 (0.526)
Median [Min, Max]	4.00 [2.01, 7.50]	3.90 [2.20, 7.50]	4.00 [2.01, 6.70]	3.90 [2.60, 7.50]	4.00 [2.01, 7.40]	3.90 [2.60, 7.50]	4.00 [2.01, 7.40]	4.00 [2.40, 7.50]	4.00 [2.01, 7.40]
Missing	16829 (75.8%)	95 (9.4%)	16734 (79.0%)	32 (16.3%)	16797 (76.4%)	8 (9.4%)	16821 (76.1%)	9 (4.4%)	16820 (76.5%)
**Troponin I**									

Mean (SD)	99.1 (1190)	201 (1820)	66.0 (888)	818 (4320)	68.8 (822)	573 (3760)	87.0 (1050)	302 (2300)	83.6 (1060)
Median [Min, Max]	0.0282 [0, 28300]	3.10 [0, 28300]	0.0115 [0, 24800]	7.76 [0, 28300]	0.0226 [0, 24800]	12.4 [0, 26300]	0.0240 [0, 28300]	4.05 [0, 26300]	0.0196 [0, 28300]
Missing	20209 (91.1%)	523 (51.8%)	19686 (92.9%)	116 (59.2%)	20093 (91.3%)	36 (42.4%)	20173 (91.3%)	65 (31.7%)	20144 (91.6%)
**Troponin T**									
Mean (SD)	0.0285 (0.107)	0.0332 (0.0596)	0.0258 (0.126)	0.0620 (0.126)	0.0272 (0.106)	0.132 (0.161)	0.0263 (0.105)	0.0856 (0.0849)	0.0268 (0.107)
Median [Min, Max]	0.00800 [0, 1.90]	0.0140 [0, 0.530]	0.00600 [0, 1.90]	0.0140 [0.00600, 0.530]	0.00800 [0, 1.90]	0.0775 [0.00800, 0.530]	0.00800 [0, 1.90]	0.0445 [0.0120, 0.257]	0.00800 [0, 1.90]
Missing	21701 (97.8%)	832 (82.4%)	20869 (98.5%)	178 (90.8%)	21523 (97.8%)	75 (88.2%)	21626 (97.8%)	191 (93.2%)	21510 (97.8%)

3. ROC curve of performance of prediction models of individual indicators : (A) full mode; (B) simplified mode

(A)

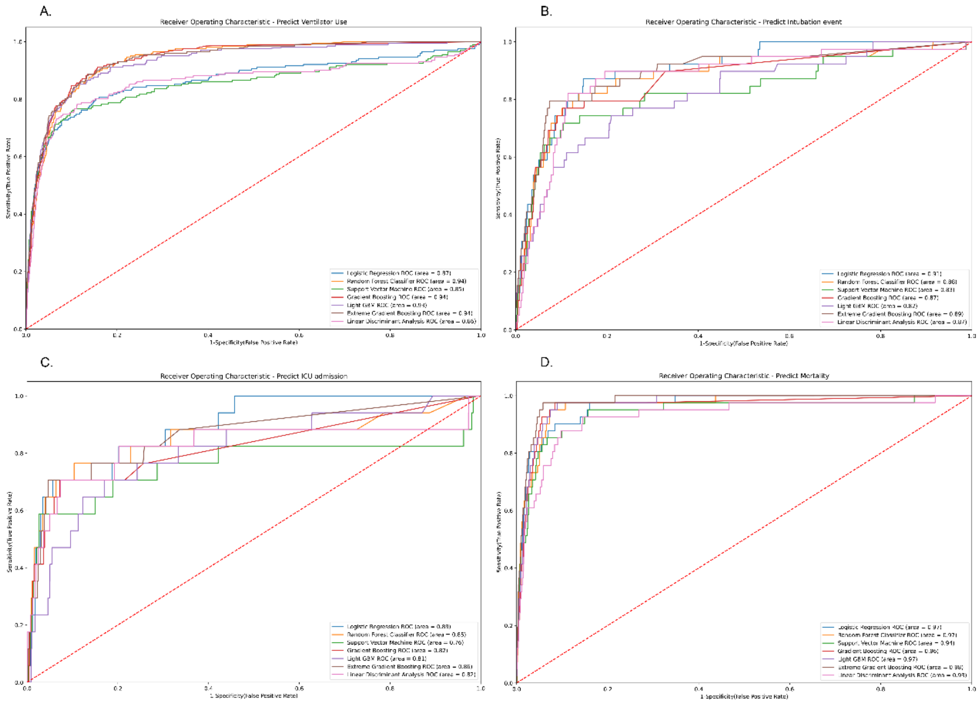

(B)

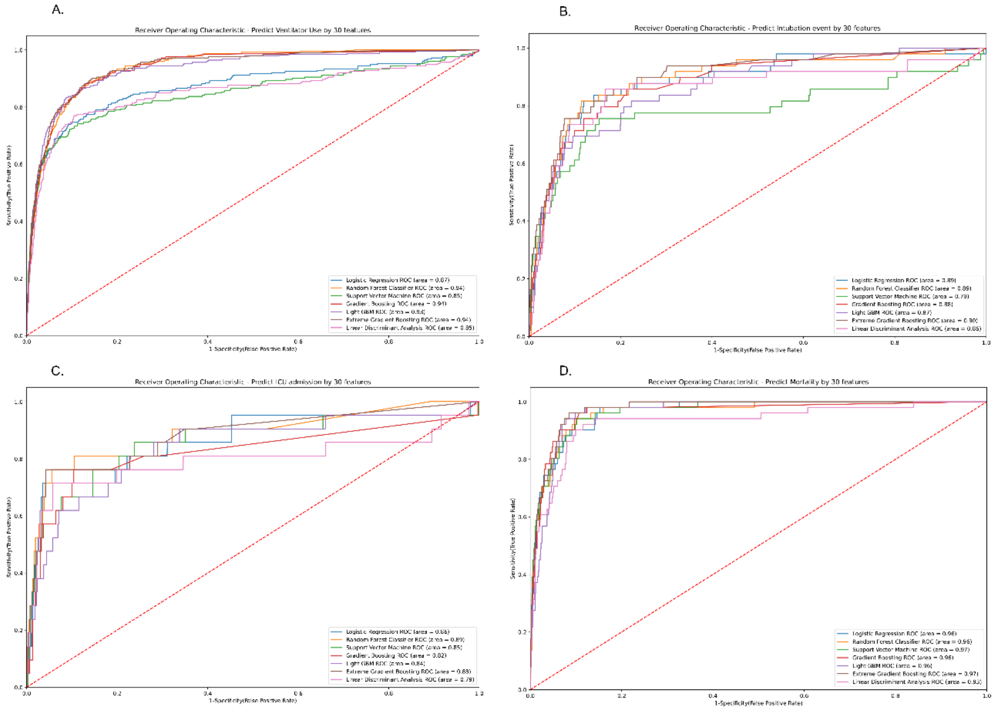

Figure 5. ROC curve of performance of prediction models of individual indicators: (A) full mode; (B) simplified mode

4. Calibration plot of performance of prediction models of severe outomes or mortality: (A) full mode; (B) simplified mode

(A)

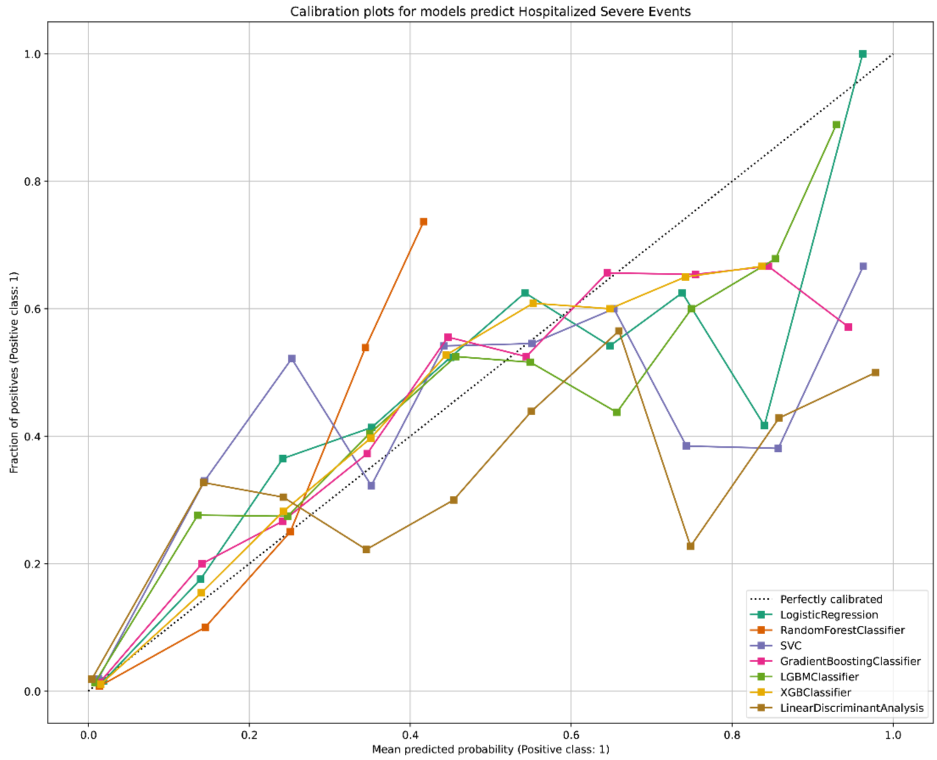

(B)

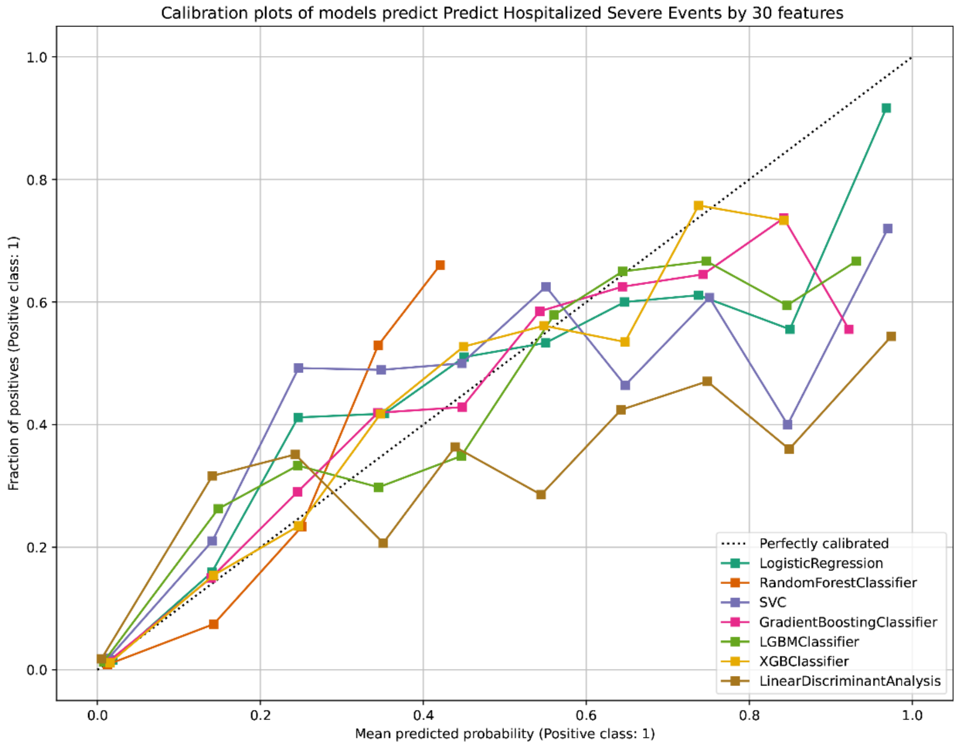

Figure 6. Calibration plot of performance of prediction models of severe outcomes or mortality: (A) full mode; (B) simplified mode

5. Calibration plot of performance of prediction models of individual indicators: (A) full mode; (B) simplified mode

(A)

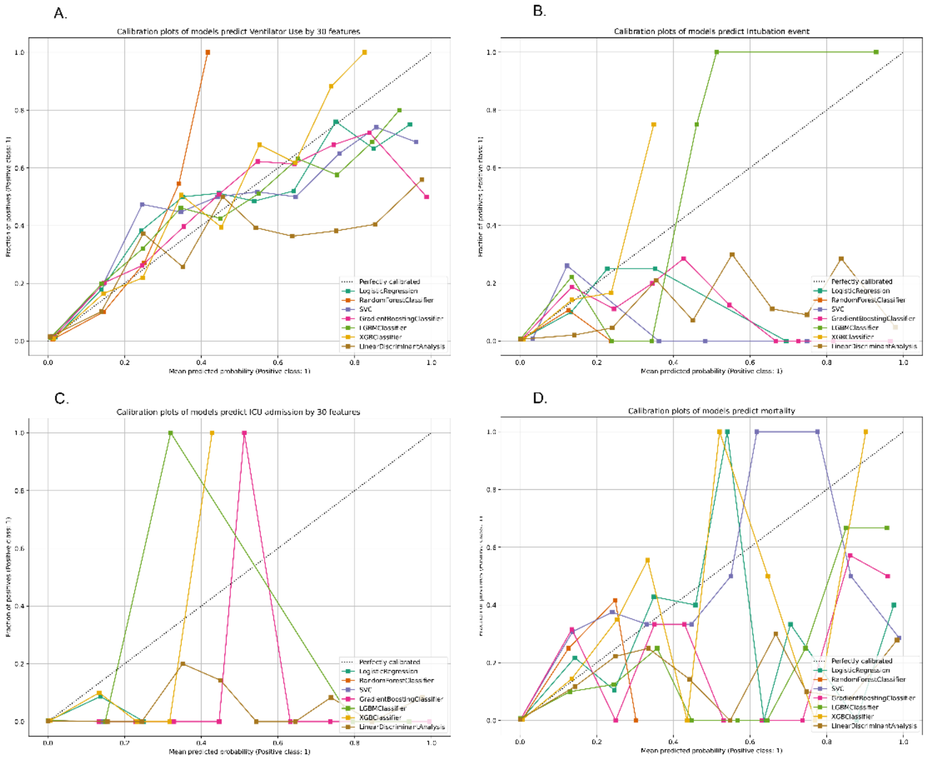

(B)

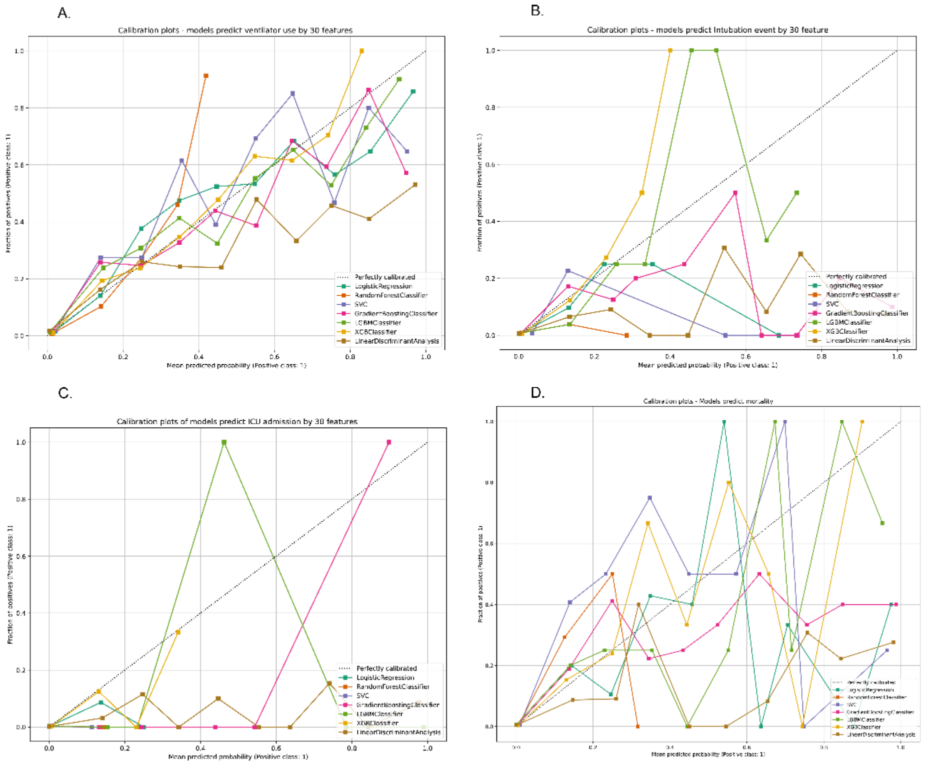

Figure 6. Calibration plot of performance of prediction models of individual indicators: (A) full mode; (B) simplified mode

6. Performance in cross-validation of prediction models on full mode

Model	Cross-validation AUC	External-validation AUC	Accuracy	Sensitivity	Specificity	PPV	NPV	F1-score
**Severe outcomes or mortality**								
Linear Discriminant Analysis	0.873	0.829	0.877	0.752	0.884	0.986	0.247	0.372
Logistic Regression	0.882	0.865	0.886	0.757	0.892	0.986	0.262	0.389
Support Vector Machine	0.869	0.823	0.837	0.743	0.841	0.985	0.192	0.305
Random Forest	0.937	0.932	0.852	0.888	0.850	0.993	0.231	0.367
Gradient Boosting	0.935	0.929	0.891	0.841	0.894	0.991	0.287	0.428
Light GBM	0.932	0.924	0.872	0.869	0.872	0.992	0.257	0.396
Extreme Gradient Boosting	0.940	0.934	0.855	0.888	0.854	0.993	0.235	0.372
**Ventilator use**								
Linear Discriminant Analysis	0.868	0.846	0.888	0.762	0.894	0.987	0.255	0.382
Logistic Regression	0.881	0.845	0.929	0.688	0.941	0.984	0.355	0.469
Support Vector Machine	0.864	0.795	0.882	0.693	0.891	0.984	0.233	0.348
Random Forest	0.934	0.930	0.857	0.881	0.856	0.993	0.226	0.360
Gradient Boosting Machine	0.932	0.924	0.855	0.876	0.854	0.993	0.222	0.354
Light GBM	0.932	0.888	0.908	0.757	0.916	0.988	0.299	0.429
Extreme Gradient Boosting	0.937	0.932	0.832	0.901	0.829	0.994	0.201	0.329
**Intubation use**								
Linear Discriminant Analysis	0.810	0.789	0.781	0.692	0.782	0.997	0.027	0.053
Logistic Regression	0.823	0.912	0.875	0.846	0.875	0.998	0.057	0.106
Support Vector Machine	0.749	0.451	0.843	0.590	0.845	0.996	0.033	0.062
Random Forest	0.890	0.870	0.789	0.846	0.788	0.998	0.034	0.066
Gradient Boosting Machine	0.864	0.820	0.790	0.795	0.790	0.998	0.033	0.063
Light GBM	0.797	0.885	0.741	0.872	0.740	0.998	0.029	0.056
Extreme Gradient Boosting	0.886	0.891	0.838	0.795	0.838	0.998	0.042	0.079
**ICU admission**								
Linear Discriminant Analysis	0.854	0.763	0.939	0.588	0.941	0.998	0.037	0.069
Logistic Regression	0.892	0.912	0.813	0.824	0.813	0.999	0.017	0.033
Support Vector Machine	0.861	0.529	0.908	0.353	0.910	0.997	0.015	0.029
Random Forest	0.952	0.843	0.780	0.824	0.780	0.999	0.014	0.028
Gradient Boosting Machine	0.907	0.633	0.908	0.353	0.910	0.997	0.015	0.029
Light GBM	0.919	0.787	0.842	0.765	0.842	0.999	0.018	0.036
Extreme Gradient Boosting	0.957	0.855	0.820	0.765	0.821	0.999	0.016	0.032
**Mortality**								
Linear Discriminant Analysis	0.914	0.924	0.818	0.927	0.817	0.999	0.045	0.086
Logistic Regression	0.959	0.959	0.951	0.854	0.952	0.999	0.142	0.243
Support Vector Machine	0.932	0.910	0.912	0.878	0.912	0.999	0.085	0.156
Random Forest	0.967	0.964	0.903	0.951	0.902	0.999	0.083	0.153
Gradient Boosting Machine	0.943	0.904	0.904	0.829	0.904	0.998	0.075	0.137
Light GBM	0.965	0.971	0.926	0.951	0.926	1.000	0.107	0.192
Extreme Gradient Boosting	0.972	0.977	0.935	0.951	0.935	1.000	0.120	0.214

7. Performance in cross-validation of prediction models on simplified mode

Model	Cross-validation AUC	External-validation AUC	Accuracy	Sensitivity	Specificity	PPV	NPV	F1-score
**Severe outcomes or mortality**								
Linear Discriminant Analysis	0.877	0.840	0.880	0.780	0.885	0.988	0.256	0.386
Logistic Regression	0.882	0.865	0.886	0.757	0.892	0.986	0.262	0.389
Support Vector Machine	0.872	0.831	0.861	0.729	0.868	0.984	0.218	0.336
Random Forest	0.938	0.932	0.875	0.864	0.875	0.992	0.259	0.399
Gradient Boosting	0.937	0.932	0.910	0.818	0.915	0.990	0.328	0.468
Light GBM	0.933	0.925	0.891	0.827	0.894	0.990	0.283	0.422
Extreme Gradient Boosting	0.941		0.878	0.860	0.879	0.992	0.264	0.404
**Ventilator use**								
Linear Discriminant Analysis	0.872	0.841	0.872	0.782	0.876	0.988	0.231	0.357
Logistic Regression	0.881	0.845	0.929	0.688	0.941	0.984	0.356	0.470
Support Vector Machine	0.869	0.800	0.910	0.673	0.921	0.983	0.289	0.405
Random Forest	0.934	0.929	0.862	0.866	0.862	0.993	0.231	0.364
Gradient Boosting Machine	0.933	0.925	0.886	0.842	0.888	0.992	0.264	0.402
Light GBM	0.932	0.885	0.892	0.762	0.898	0.988	0.263	0.391
Extreme Gradient Boosting	0.937	0.932	0.831	0.896	0.828	0.994	0.199	0.326
	0.933	0.925	0.886	0.842	0.888	0.992	0.264	0.402
**Intubation use**								
Linear Discriminant Analysis	0.817	0.840	0.818	0.718	0.819	0.997	0.034	0.065
Logistic Regression	0.822	0.912	0.875	0.846	0.875	0.998	0.057	0.106
Support Vector Machine	0.757	0.831	0.760	0.769	0.760	0.997	0.028	0.053
Random Forest	0.890	0.876	0.770	0.846	0.770	0.998	0.032	0.061
Gradient Boosting Machine	0.861	0.782	0.846	0.692	0.847	0.997	0.039	0.073
Light GBM	0.817	0.877	0.774	0.846	0.774	0.998	0.032	0.062
Extreme Gradient Boosting	0.885	0.890	0.853	0.795	0.854	0.998	0.046	0.087
**ICU admission**								
Linear Discriminant Analysis	0.862	0.742	0.959	0.529	0.960	0.998	0.049	0.089
Logistic Regression	0.892	0.912	0.813	0.824	0.813	0.999	0.017	0.033
Support Vector Machine	0.833	0.544	0.877	0.647	0.878	0.998	0.020	0.039
Random Forest	0.955	0.846	0.895	0.706	0.896	0.999	0.025	0.049
Gradient Boosting Machine	0.915	0.584	0.991	0.059	0.995	0.996	0.043	0.050
Light GBM	0.941	0.810	0.831	0.765	0.831	0.999	0.017	0.033
Extreme Gradient Boosting	0.958	0.857	0.760	0.824	0.760	0.999	0.013	0.026
**Mortality**								
Linear Discriminant Analysis	0.925	0.947	0.846	0.927	0.846	0.999	0.053	0.100
Logistic Regression	0.959	0.959	0.951	0.854	0.952	0.999	0.142	0.243
Support Vector Machine	0.953	0.937	0.825	0.927	0.824	0.999	0.047	0.089
Random Forest	0.967	0.953	0.901	0.951	0.901	0.999	0.082	0.151
Gradient Boosting Machine	0.964	0.896	0.888	0.878	0.888	0.999	0.068	0.127
Light GBM	0.962	0.962	0.938	0.951	0.938	1.000	0.125	0.221
Extreme Gradient Boosting	0.970	0.975	0.943	0.927	0.943	0.999	0.131	0.230

8. ROC curve of cross – validation performance of the prediction models

(A)

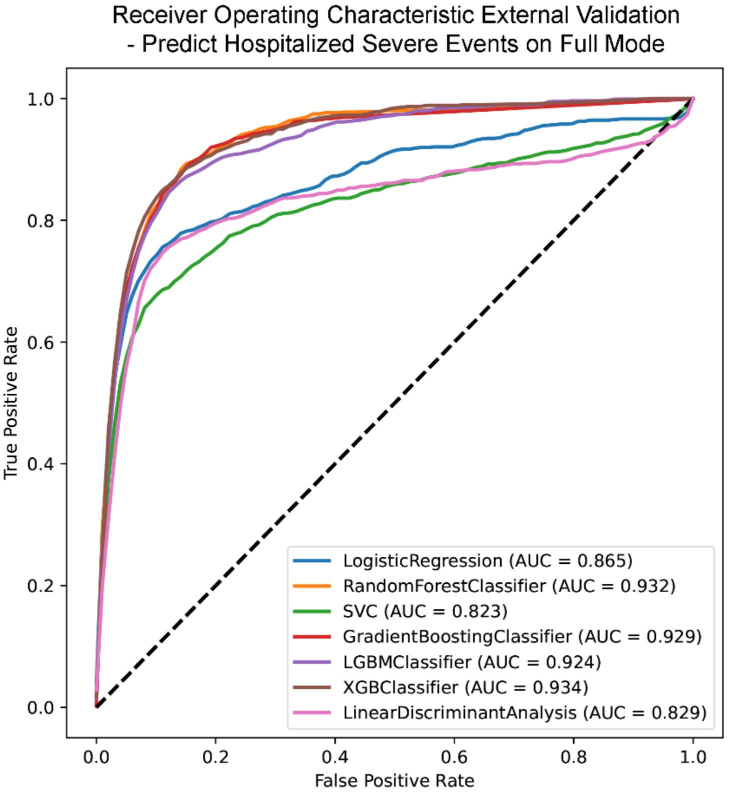

(B)

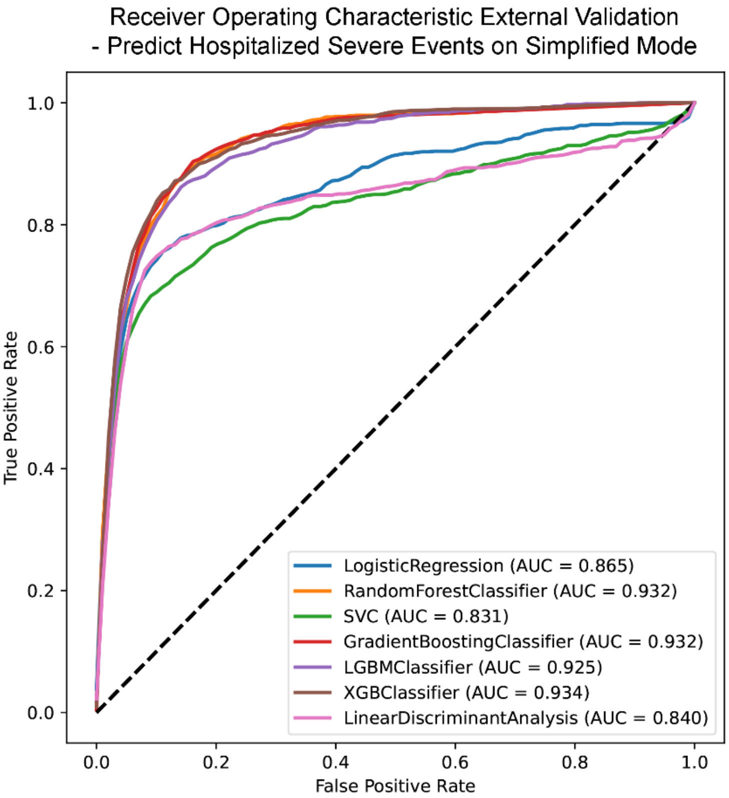

Figure 7. ROC curve of cross – validation performance of prediction models of models of severe outomes or mortality: (A) full mode; (B) simplified mode

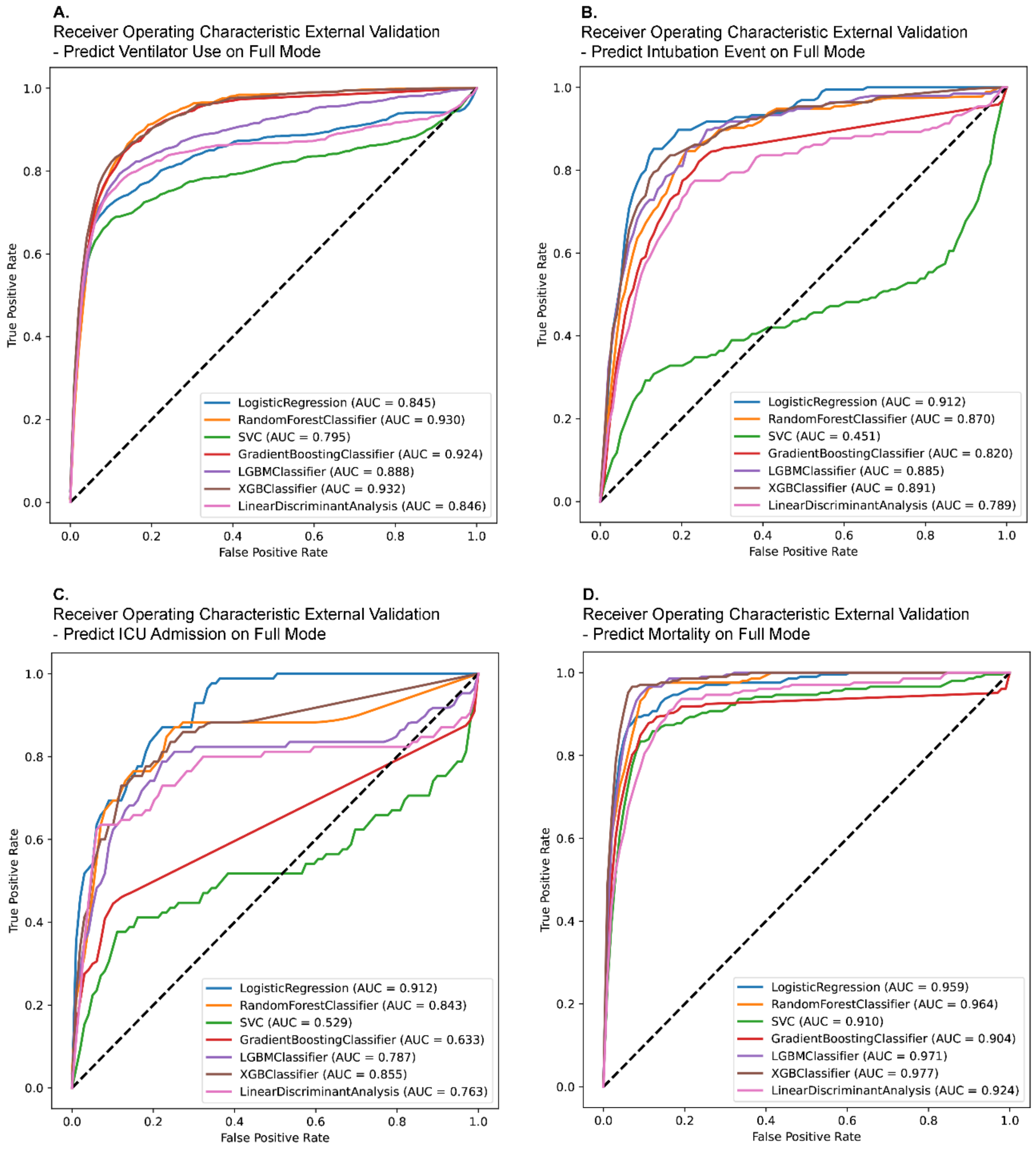

Figure 8. ROC curve of performance of prediction models of individual indicators on full mode

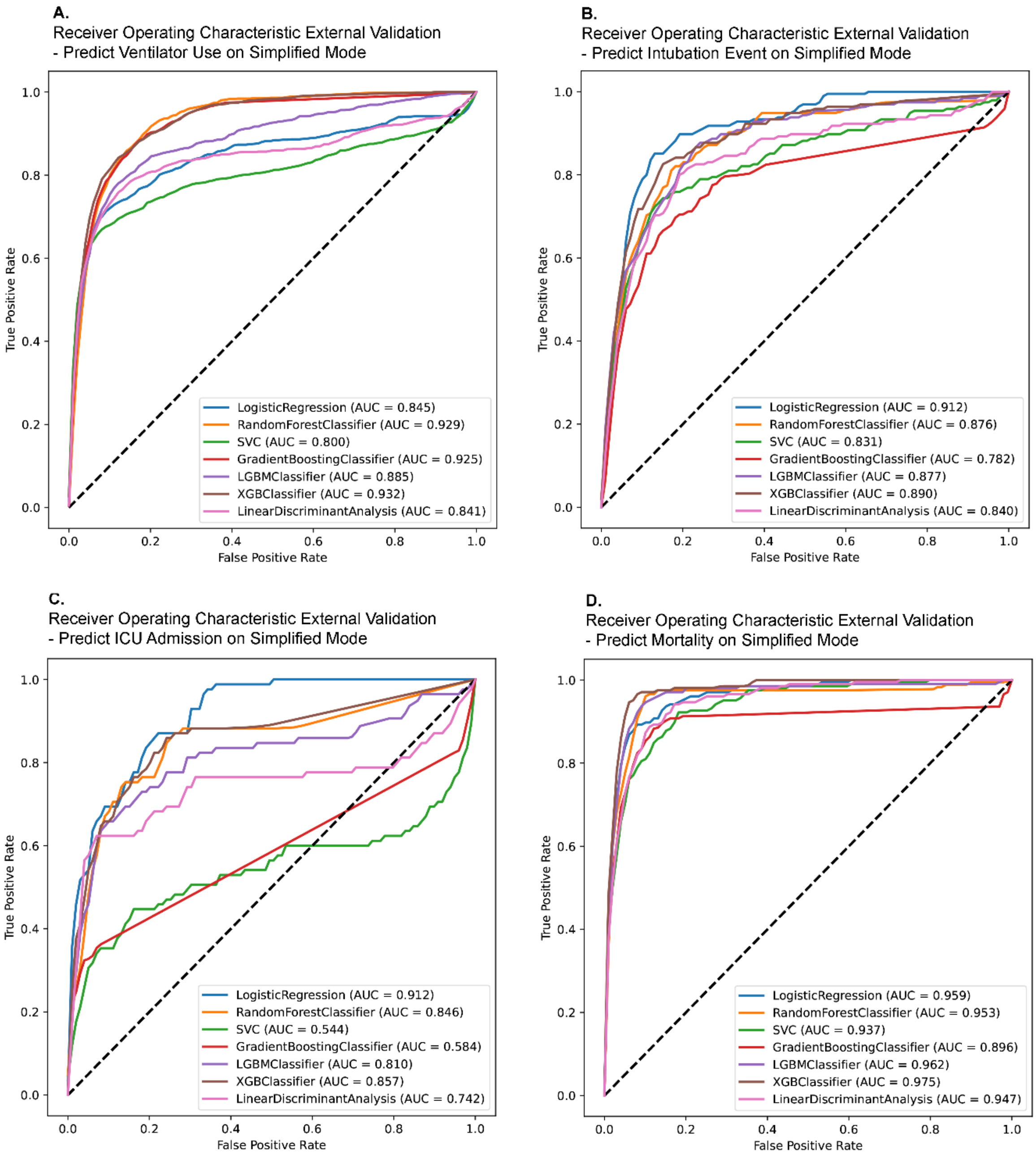

Figure 9. ROC curve of performance of prediction models of individual indicators on simplified mode

9. STROBE Statement

	Item No	Recommendation	Page No
**Title and abstract**	1	(*a*) Indicate the study's design with a commonly used term in the title or the abstract<br>(*b*) Provide in the abstract an informative and balanced summary of what was done and what was found	1<br>2
**Introduction**			
Background/rationale	2	Explain the scientific background and rationale for the investigation being reported	3
Objectives	3	State specific objectives, including any prespecified hypotheses	3
**Methods**			
Study design	4	Present key elements of study design early in the paper	3
Setting	5	Describe the setting, locations, and relevant dates, including periods of recruitment, exposure, follow-up, and data collection	3
Participants	6	(*a*) Give the eligibility criteria, and the sources and methods of selection of participants. Describe methods of follow-up<br>(*b*) For matched studies, give matching criteria and number of exposed and unexposed	3
Variables	7	Clearly define all outcomes, exposures, predictors, potential confounders, and effect modifiers. Give diagnostic criteria, if applicable	4-5
Data sources/ measurement	8*	For each variable of interest, give sources of data and details of methods of assessment (measurement). Describe comparability of assessment methods if there is more than one group	3
Bias	9	Describe any efforts to address potential sources of bias	5
Study size	10	Explain how the study size was arrived at	
Quantitative variables	11	Explain how quantitative variables were handled in the analyses. If applicable, describe which groupings were chosen and why	4-5
Statistical methods	12	(*a*) Describe all statistical methods, including those used to control for confounding<br>(*b*) Describe any methods used to examine subgroups and interactions<br>(*c*) Explain how missing data were addressed<br>(*d*) If applicable, explain how loss to follow-up was addressed<br>(*e*) Describe any sensitivity analyses	5-6<br>5-6<br>4<br>NA<br>NA
**Results**			
Participants	13*	(a) Report numbers of individuals at each stage of study—eg numbers potentially eligible, examined for eligibility,	6

		confirmed eligible, included in the study, completing follow-up, and analysed<br>(b) Give reasons for non-participation at each stage<br>(c) Consider use of a flow diagram	
Descriptive data	14*	(a) Give characteristics of study participants (eg demographic, clinical, social) and information on exposures and potential confounders<br>(b) Indicate number of participants with missing data for each variable of interest<br>(c) Summarise follow-up time (eg, average and total amount)	6<br>6<br>NA
Outcome data	15*	Report numbers of outcome events or summary measures over time	6-7

Reference

1. Balakrishnama S, Ganapathiraju A. Linear discriminant analysis-a brief tutorial. Institute for Signal and information Processing. 1998;18(1998):1-8.
2. POORHAJI MM, SHAHRIYARI M, ASADI MM, BAHRAMI F, GOUVRCHINGHALEH HE, MOHAMMADZADEH T, et al. Binary Logistic Regression and Linear Discriminant Analyses in Evaluating Laboratory Factors Associated with COVID-19: A Comparison of Two Statistical Methods. Mediterranean Journal of Infection, Microbes & Antimicrobials. 2022;11.
3. Rao CR. The utilization of multiple measurements in problems of biological classification. Journal of the Royal Statistical Society Series B (Methodological). 1948;10(2):159-203.
4. Maalouf M. Logistic regression in data analysis: an overview. International Journal of Data Analysis Techniques and Strategies. 2011;3(3):281-99.
5. Park H-A. An introduction to logistic regression: from basic concepts to interpretation with particular attention to nursing domain. Journal of Korean Academy of Nursing. 2013;43(2):154-64.
6. Boateng EY, Abaye DA. A review of the logistic regression model with emphasis on medical research. Journal of data analysis and information processing. 2019;7(4):190-207.
7. Bisong E, Bisong E. Logistic regression. Building Machine Learning and Deep Learning Models on Google Cloud Platform: A Comprehensive Guide for Beginners. 2019:243-50.
8. Huang S, Cai N, Pacheco PP, Narrandes S, Wang Y, Xu W. Applications of support vector machine (SVM) learning in cancer genomics. Cancer genomics & proteomics. 2018;15(1):41-51.
9. Jakkula V. Tutorial on support vector machine (svm). School of EECS, Washington State University. 2006;37(2.5):3.
10. Breiman L. Random forests. Machine learning. 2001;45:5-32.
11. Osi AA, Abdu M, Muhammad U, Ibrahim A, Isma'il LA, Suleiman AA, et al. A classification approach for predicting COVID-19 Patient's survival outcome with machine learning techniques. MedRxiv. 2020:2020.08. 02.20129767.
12. Bentéjac C, Csörgő A, Martínez-Muñoz G. A comparative analysis of gradient boosting algorithms. Artificial Intelligence Review. 2021;54:1937-67.
13. Ramón A, Torres AM, Milara J, Cascón J, Blasco P, Mateo J. eXtreme Gradient Boosting-based method to classify patients with COVID-19. Journal of Investigative Medicine. 2022;70(7):1472-80.
14. Ernawan F, Handayani K, Fakhreldin M, Abbker Y. Light Gradient Boosting with Hyper Parameter Tuning Optimization for COVID-19 Prediction. International Journal of Advanced Computer Science and Applications. 2022;13(8).
15. Chen T, He T, Benesty M, Khotilovich V, Tang Y, Cho H, et al. Xgboost: extreme gradient boosting. R package version 04-2. 2015;1(4):1-4.
